# Deep-water lymnaeid gastropods (Hygrophila) of Lake Pannon (Late Miocene, Central Europe): taxonomy and biostratigraphic significance

**DOI:** 10.3897/zookeys.1282.186967

**Published:** 2026-06-12

**Authors:** Dániel Botka, Imre Magyar

**Affiliations:** 1 Laboratories MOL, MOL Plc., Szolnok, 5000, Hungary Department of Palaeontology, Institute of Geography and Earth Sciences, Eötvös Loránd University Budapest Hungary; 2 Department of Palaeontology, Institute of Geography and Earth Sciences, Eötvös Loránd University, Budapest, 1117, Hungary Hungarian National Museum Public Collection Centre, Budapest – Hungarian Natural History Museum Budapest Hungary; 3 Exploration and Production, MOL Plc., Budapest, 1117, Hungary Laboratories MOL, MOL Plc. Szolnok Hungary; 4 HUN-REN–MTM–ELTE Research Group for Paleontology, Budapest, Hungary Exploration and Production, MOL Plc. Budapest Hungary; 5 Hungarian National Museum Public Collection Centre, Budapest – Hungarian Natural History Museum, Budapest, 1083, Hungary HUN-REN–MTM–ELTE Research Group for Paleontology Budapest Hungary

**Keywords:** Anacladogenesis, brackish-water, convergent evolution, Lymnaeidae, Pannonian Basin

## Abstract

In the present study, a distinctive lineage of lymnaeid gastropods from the deep-water deposits of the Late Miocene Lake Pannon (Central Europe) is revised. The examined material includes specimens from museum collections as well as newly collected fossils from different parts of the Pannonian Basin System. During the past 150 years, at least 30 deep-water lymnaeid species assigned to ten genera have been described from the Lake Pannon deposits; however, our examination of more than 700 specimens shows that these records represent only 11 species belonging to five genera. Lectotypes are designated for four species for which earlier type designations were invalid. Species belonging to the *Radix*–*Velutinopsis*–*Undulotheca*–*Provalenciennesia*–*Valenciennius* lineage form one of the most extraordinary molluscan groups of Lake Pannon. Their interest lies in their distinctive shell morphology – large (sometimes exceeding 10 cm), wide, thin, cap-like shells with reduced coiling and undulated ribs – and their deep-water mode of life, supported by occurrences in drill cores and bathymetrically calibrated by seismic sections. This taxonomic revision enables the lineage to be applied in deep-water biostratigraphy of Lake Pannon sediments. Based on first appearance datums of key lineage species and associated taxa from other mollusc groups, four new lineage zones are proposed, integrated with the established deep-water mollusc biozonation. In contrast to the anagenetic evolutionary patterns typical of biostratigraphically important Lake Pannon bivalves, this gastropod lineage shows predominantly anacladogenetic speciation.

## Introduction

Freshwater lymnaeid gastropods (Lymnaeidae, Hygrophila) are widely distributed worldwide, with the highest diversity in North America and Eurasia (Aksenova et al. 2018; Vinarski et al. 2019, 2023). They exhibit extensive shell shape variation, ranging from strongly sculptured morphotypes to ancyliform (limpet-like) forms (Vinarski and Pointier 2023). Lymnaeids are common components of freshwater ecosystems, occurring across a broad spectrum of aquatic habitats, and their shells are abundant in the fossil record, with an estimated 370–380 valid species from pre-Pleistocene times (Wenz 1923; Neubauer 2023, 2024). This rich fossil record makes them valuable palaeoecological indicators (e.g. Salvador et al. 2018; Rasser et al. 2020; Kadolsky 2020). Although extant lymnaeids are often classified as strictly freshwater molluscs, some taxa tolerate elevated salinity levels. Examples compiled by Vinarski and Pointier (2023) include live Baltic Sea lymnaeids at salinities of ≥2‰ (Carlsson 2006), *Radix obliquata* in Issyk-Kul Lake, Central Asia, Kyrgyzstan at 5.8–5.9‰ (Pavlova 1983), and several species inhabiting mineralised lakes in northern Kazakhstan at 4.95‰ (Andreeva et al. 2010). The water depth limit tolerated by lymnaeid snails, however, appears to be no greater than 300 m based on recent analogues. The deepest known populations of these molluscs were discovered in alpine lakes in Switzerland and S Germany (e.g. Clessin 1877). Even so, these records are modest compared with those of some other aquatic pulmonates, such as Lake Baikal endemic acroloxid snails that have been recorded from depths up to 1000 m (Shirokaya and Takhteev 2018). In most large lakes, however, Lymnaeidae are largely confined to the shallow littoral zone and usually occur within the first few meters below the surface; genuinely deep-water occurrences are uncommon (Vinarski and Aksenova 2023).

Long-lived lakes frequently foster intra-lacustrine adaptive radiations and therefore contribute disproportionately to the global diversity of fossil freshwater and brackish-water gastropods. Lake Pannon in Central Europe, for example, accounts for 11.2% of all recorded fossil freshwater and brackish-water gastropod species, with 579 species documented (Neubauer et al. 2016; Neubauer 2024).

Lake Pannon formed after isolation from the Central Paratethys Sea at the beginning of the Late Miocene period – ca 11.6 Ma ago – within the intra-Carpathian realm. This vast, long-lived brackish-water lake persisted for ca 8 Ma and accumulated a thick sedimentary infill. Its deposits are widely distributed and can be studied across ten present-day countries in Central Europe. Exploration for geoenergy resources within these sediments stimulated the development of a regional stratigraphic framework that is based largely on the evolutionary succession of endemic bivalves and dinoflagellates. Mollusc species in Lake Pannon evolved from both marine relict and freshwater immigrant lineages (Müller et al. 1999; Magyar et al. 1999a, 1999b, 2025; Magyar and Geary 2012; Neubauer et al. 2016).

More than 50 lymnaeid species have been described from the primarily brackish-water Upper Miocene to Pliocene deposits of the Central and Eastern Paratethyan realms, including the Pannonian Basin (Lake Pannon) and the Dacian, Black Sea, and Caspian basins (Eastern Paratethys). These taxa likely tolerated salinities higher than typical freshwater conditions (e.g. 8–15‰ in Lake Pannon; Müller et al. 1999). Most species were endemic to their respective water bodies; however, Michel (1994) reported that only 11% of extant long-lived lake lymnaeids are endemic.

The biostratigraphic potential of Lake Pannon lymnaeids has not been fully exploited, largely because of their mosaic stratigraphic record, generally poor preservation, and frequent misidentifications in the literature. Nevertheless, some early biostratigraphic or taxonomic efforts addressed Lake Pannon taxa (Gorjanović-Kramberger 1901, 1923; Moos 1944) as well as Eastern Paratethyan forms (Taktakishvili 1967). The history of these lymnaeids did not end with the infilling of Lake Pannon, they successfully migrated into the Eastern Paratethyan region (Dacian and Euxinic basins), where their radiation is documented from the upper Maeotian and Pontian (Late Miocene – Messinian) until the Dacian/Kimmerian and lower Romanian/lower Kuyalnikian strata (Pliocene – Zanclean and Piacenzian) (Taktakishvili 1967; Marinescu 1969; Popov et al. 2022). These Eastern Paratethyan forms may represent direct descendants of the Lake Pannon lineage; however, this hypothesis can only be evaluated through a careful taxonomic revision of the Eastern Paratethyan material, which is beyond the scope of the present study. At least 30 deep-water lymnaeid species assigned to ten genera have been described from the Lake Pannon deposits attributed to the *Radix*–*Velutinopsis*–*Undulotheca*–*Provalenciennesia*–*Valenciennius* lineage. Many of these species were erected on the basis of minor differences, non-diagnostic characters, or differently compacted and/or poorly preserved specimens, likely inflating the reported species richness (Gorjanović-Kramberger 1901, 1923; Moos 1944; Kochansky-Devidé and Pikija 1976). In addition, Kochansky-Devidé and Pikija (1976) and Sremac (1981) introduced the genera *Neodelminiella* and *Neoclivunella* from Lake Pannon sediments (northern Croatia), citing similarities (limpet-like shell and reduced coiling) to the freshwater Early to Middle Miocene *Delminiella* and *Clivunella* species known from the Dinaride Lake System and from the paleolake Lavant in the Eastern Alps, Austria (Kochansky-Devidé and Slišković 1972; Harzhauser et al. 2016).

The aim of the present study is to revise the Lake Pannon taxa and to provide detailed descriptions and photographic documentation of the available material, including type materials and newly collected specimens. Their biostratigraphic relevance within Lake Pannon biozonation is also discussed. We here clarify the type status of selected Lake Pannon lymnaeid taxa and designate new lectotypes for four species (*Provalenciennesia limnaeoidea* (Gorjanović-Kramberger, 1901), *P. arthaberi* (Gorjanović-Kramberger, 1901), *P. boeckhi* (Halaváts, 1886), *Valenciennius kiseljaki* Gorjanović-Kramberger, 1901) where previous type designations were invalid or absent.

## Materials and methods

This study includes the members of the brackish, deep-water Pannonian (Late Miocene) *Radix*–*Velutinopsis*–*Undulotheca*–*Provalenciennesia*–*Valenciennius* evolutionary lineage. Other shallow-water Lake Pannon lymnaeids are not part of this study. The studied material comprises lymnaeid gastropod fossils of 12 museum collections from Hungary, Croatia, Serbia, Austria, Romania, and France (see the list of collections in the Abbreviations). Additional specimens were recently collected by the authors of this paper. The fossils collected by the authors were cleaned and prepared in the laboratory of the Department of Palaeontology at the Eötvös Loránd University, Budapest and in the Natural History Museum, Budapest. Polyvinyl acetate was used for solidifying fragile fossils. For comparison of specimens, all the available type materials were studied in the mentioned museum collections. Altogether, 713 specimens were studied in detail, of which 552 specimens were determined to species level. Synonyms, original and emended diagnoses, type localities, type materials, studied specimens, photos, measurements, geographical distribution data, and stratigraphic range of the given species, and remarks on the earlier questionable identifications are provided in the paper. Geographic names are given according to the present-day usage, but sometimes other older (Hungarian and/or German) names are also indicated to help the identification of old localities. All data of the studied specimens can be found in Suppl. material 1.

### Abbreviations

**SARA** Department of Collections, Supervisory Authority for Regulatory Affairs/Szabályozott Tevékenységek Felügyeleti Hatósága (former MÁFI/GIH – Magyar Állami Földtani Intézet/Geological Institute of Hungary), Budapest, Hungary

**HNHM** Hungarian Natural History Museum/Magyar Természettudományi Múzeum, Budapest, Hungary

**ELTE** Palaeontological Collection of the Department of Palaeontology of the Eötvös Loránd University, Budapest, Hungary

**CNHM** Croatian Natural History Museum/Hrvatski prirodoslovni muzej, Zagreb, Croatia

**NHMB** Natural History Museum Belgrade/Prirodnjački muzej Beograd, Belgrade, Serbia

**GS** GeoSphere Austria (former Geologische Bundesanstalt), Vienna, Austria

**NHMV** Natural History Museum Vienna/Naturhistorisches Museum Wien, Vienna, Austria

**BBU** Babeş-Bolyai University, Cluj-Napoca/Kolozsvár, Romania

**NGM-GIR** National Geology Museum of the Geological Institute of Romania, Bucharest, Romania

**BM** Brukenthal Museum, Sibiu/Nagyszeben/Hermannstadt, Romania

**MIM** Molnár István Museum, Cristuru Secuiesc/Székelykeresztúr, Romania

**UL** Collections de Paléontologie, Laboratoire de Géologie de Lyon, Université Lyon, Lyon, France

**L** – length; **W** – width; **L/W** – length/width ratio; **LBw** – length of body whorl; **NR** – number of ribs; **WR** – width of ribs; **WSR** – width of the space between ribs; **FAD** – first appearance datum; **LAD** – last appearance datum; * – first mention of the accepted species name in the synonym lists; ***1970*** – synonyms without figured specimens

## Results

### Systematic palaeontology

**Lymnaeidae Rafinesque, 1815**

#### Amphipepleinae Pini, 1877

##### Genus Radix

Taxon classificationAnimaliaLymnaeidaLymnaeidae

Montfort, 1810

6A002A04-B3BC-5297-9D22-762125CE71DB

###### Type species.

*Radix auricularia* (Linnaeus, 1758); original designation.

###### Diagnosis.

“Shell shape varies from auriculate to high conical, the body whorl and the aperture are usually well developed. The spire is usually short and acute.” (Aksenova et al. 2018).

##### Radix croatica

Taxon classificationAnimaliaLymnaeidaLymnaeidae

(Gorjanović-Kramberger, 1890)

D8CE18A0-E29F-567B-B921-DF7A247D7CBB

Fig. 1A–E

**Figure 1.**
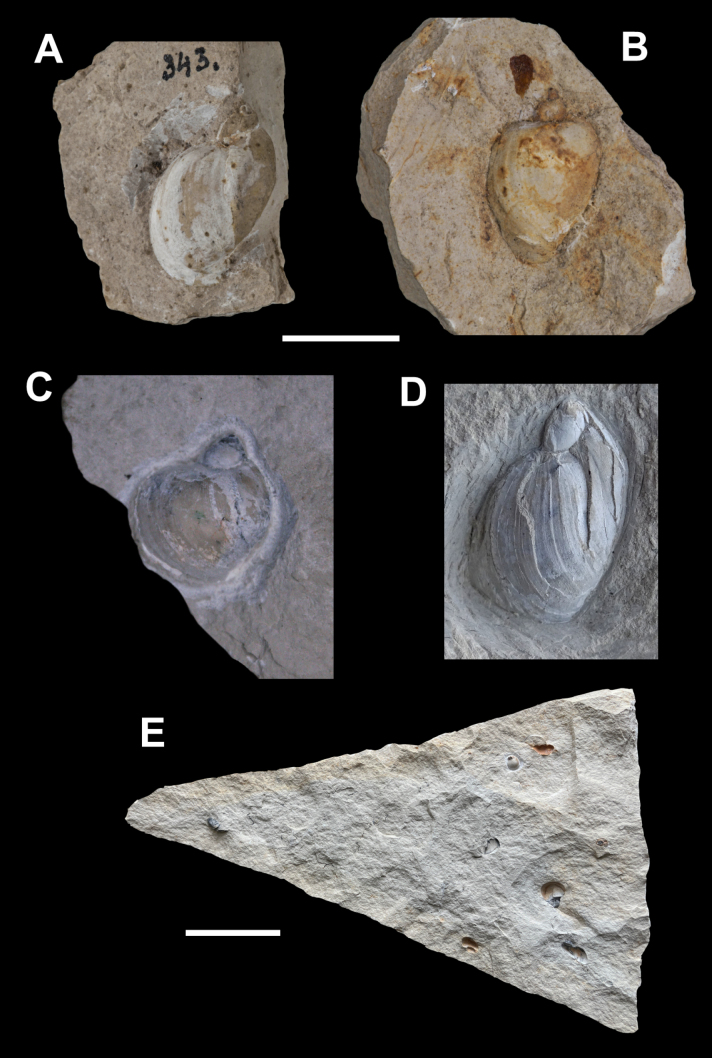
**A–E**. *Radix croatica* (Gorjanović-Kramberger, 1890). **A**. Syntype specimen, Moravče, CNHM 5178-343, photo: Nives Borčić; **B**. Syntype specimen, Moravče, CNHM 5178-344, photo: Nives Borčić; **C**. Našice/Nekcse, HNHM INV 2025.205; **D**. Hidas-53 borehole, HNHM INV 2025.186; **E**. Platy limestone lamina with lymnaeid and planorbid steinkerns typical for the “Croatica beds”, Našice/Nekcse, HNHM INV 2025.207. Scale bars: 1 cm (**A–D**), 5 cm (**E**).

**Figure 2.**
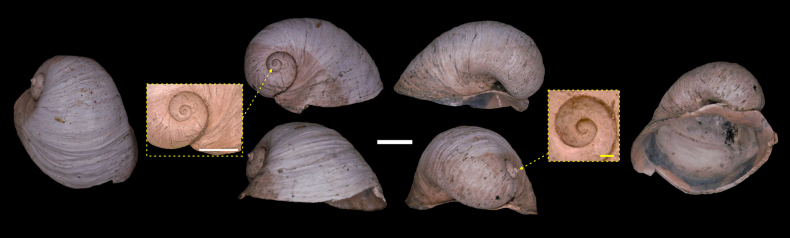
*Velutinopsis velutina* (Deshayes, 1838), Bátaszék, HNHM INV 2025.179. Scale bars: 1 cm (white), 1 mm (yellow).

**Figure 3.**
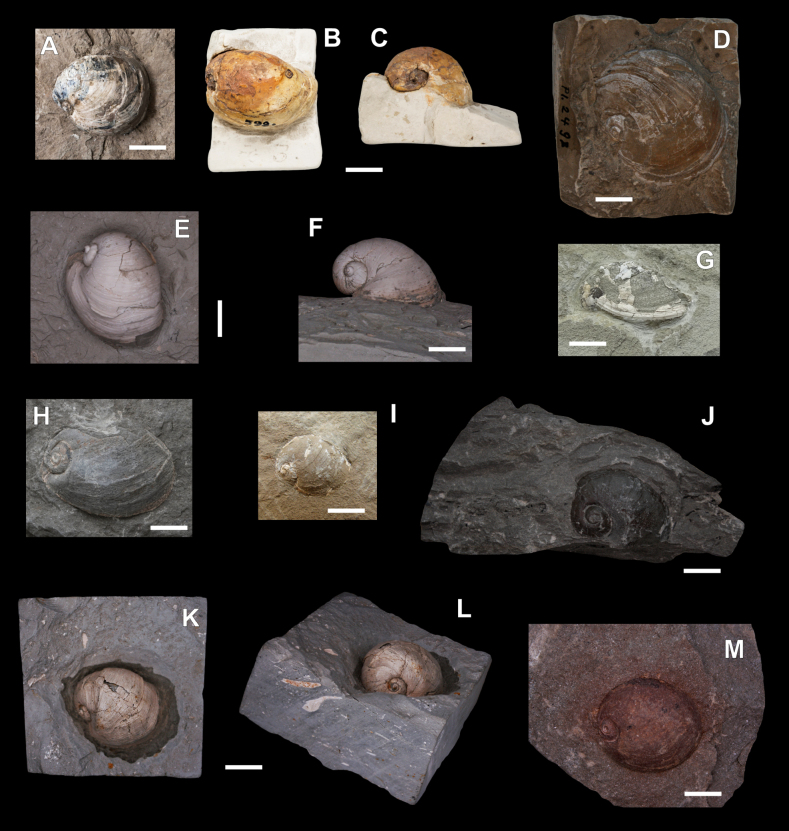
*Velutinopsis velutina* (Deshayes, 1838). **A**. Peklenica-47 borehole, CNHM 7, photo: Nives Borčić; **B, C**. holotype specimen of *Velutinopsis simplex* (Gorjanović-Kramberger, 1899), Londžica/Babindol, CNHM 5449-599, photo: Nives Borčić; **D**. Beočin/Beocsin/Belcsény, SARA Pl.2493; **E, F**. Bátaszék, HNHM INV 2025.174; **G**. Gușterița/Szenterzsébet/Hammersdorf, HNHM INV 2025.245; **H**. Ludbreg-1 borehole, CNHM 2, photo: Nives Borčić; **I**. Juvenile specimen, Mihalț/Mihálcfalva, HNHM INV 2025.234; **J**. Nagykörű-3 borehole, SARA Pl.2024.100.1; **K, L**. Bátaszék, HNHM INV 2025.175; **M**. Őriszentpéter-1 borehole, SARA Pl.2024.35.1. Scale bars: 1 cm.

**Figure 4.**
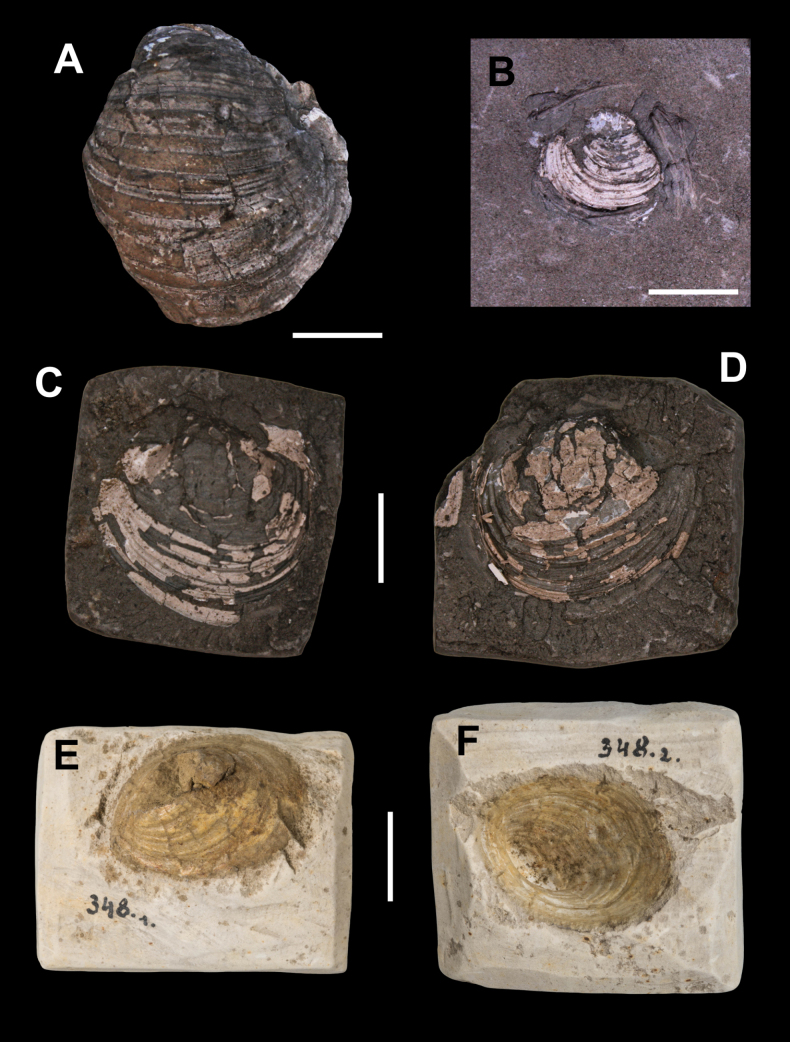
*Velutinopsis rugosa* (Gorjanović-Kramberger, 1901). **A**. Holotype specimen, Beočin/Beocsin/Belcsény, SARA Pl.2497; **B**. Juvenile specimen, Sângeorgiu de Mureș/Marosszentgyörgy, SARA Pl.2024.628.1; **C, D**. Tătârlaua/Felsőtatárlaka, SARA Pl.2024.377.1; **E, F**. Lectotype specimen of *Hiscerus amplectus* (Gorjanović-Kramberger, 1901), Zagreb-Gračani, CNHM 5183-348/1-2, photo: Nives Borčić. Scale bars: 1 cm.

**Figure 5.**
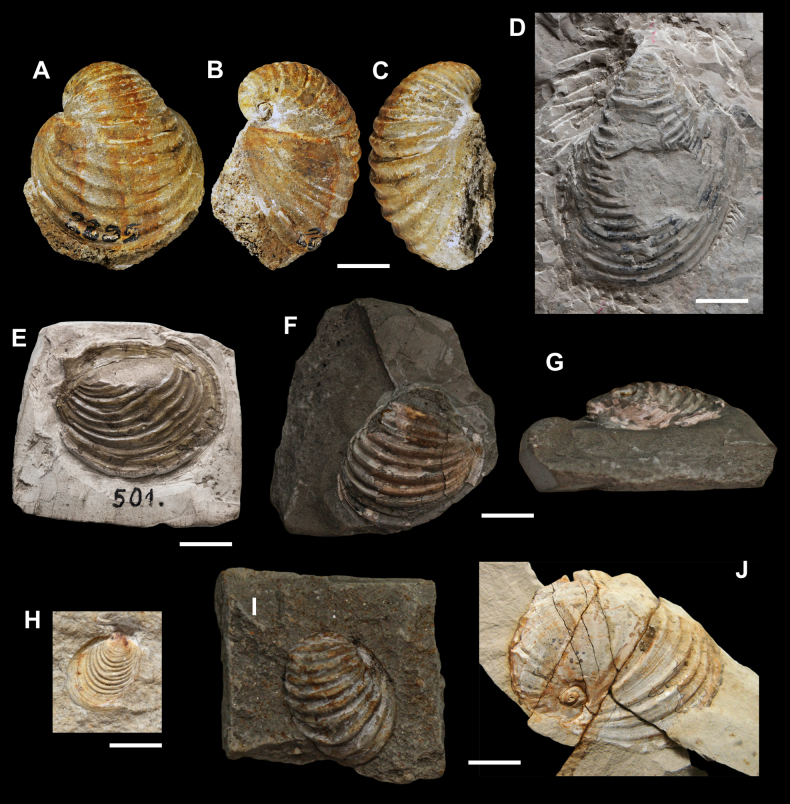
*Undulotheca nobilis* (Reuss, 1868). **A–C**. Lectotype specimen, Şura Mare/Nagycsűr–Hamba/Kakasfalva/Hahnbach, GS 1868/005/0001/1, photo: Lajos Katona; **D**. Holotype specimen of *Velutinopsis transiens* Moos, 1944, Grubišno Polje-1 borehole, CNHM 12, photo: Nives Borčić; **E**. holotype+genotype specimen of *Hiscerus undulatus* (Gorjanović-Kramberger, 1901), Novi Marof, CNHM 5348-501, photo: Nives Borčić; **F, G**. Axente Sever/Asszonyfalva, SARA Pl.1811; **H**. Juvenile holotype+genotype specimen of *Neodelminiella venusta* Kochansky-Devidé & Pikija, 1976, Zagreb–Vugrovec, CNHM 983, photo: Nives Borčić; **I**. Slimnic/Szelindek, SARA Pl.1800. J: Oarba de Mureș/Marosorbó, HNHM INV 2025.228. Scale bars: 1 cm.

**Figure 6.**
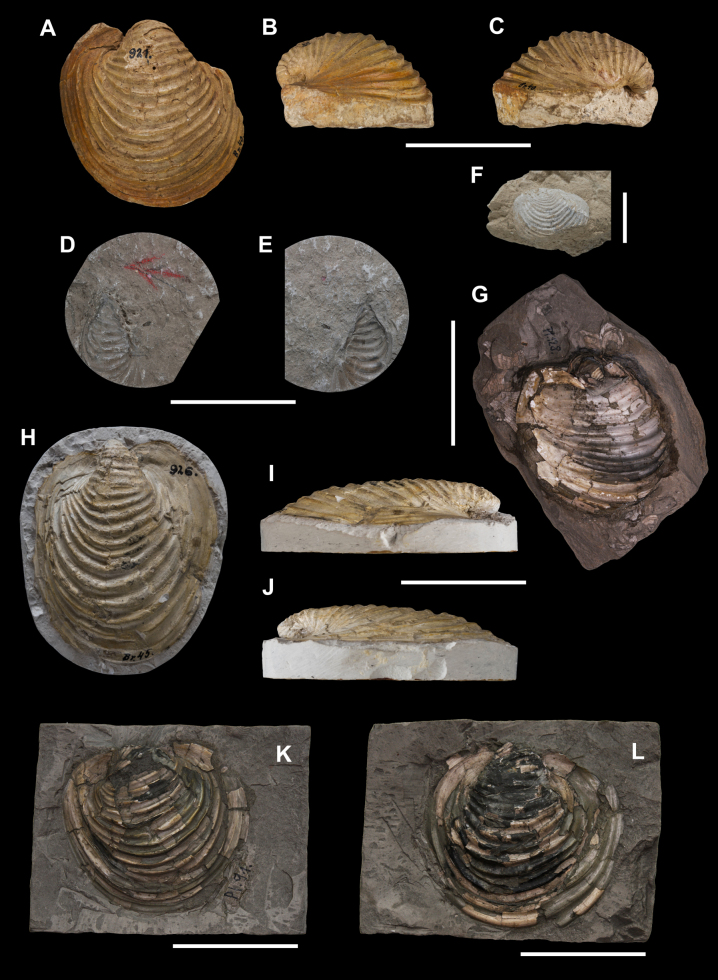
*Undulotheca pancici* (Brusina, 1893). **A–C**. holotype specimen of *Undulotheca pancici* (Brusina, 1893), Zagreb–Markuševec, CNHM 5771-921, photo: Nives Borčić; **D, E**. Holotype specimen of *Undulotheca gojlo* Moos, 1944, Gojlo-4 borehole, CNHM 18/1-2, photo: Nives Borčić; **F**. Juvenile holotype specimen of *Neodelminiella lucinoides* Kochansky-Devidé & Pikija, 1976, Voćarica, CNHM 984, photo: Nives Borčić; **G**. Holotype specimen of *Undulotheca halavatsi* (Gorjanović-Kramberger, 1901), Vârciorova/Varcsaró, SARA Pl.128; **H–J**. holotype specimen of *Undulotheca kochi* Gorjanović-Kramberger, 1923, Bektež, CNHM 5776-926, photo: Nives Borčić; **K–L**. Vârciorova/Varcsaró, SARA Pl.91, SARA Pl.6370. Scale bars: 5 cm (**A–E, G–L**), 2 cm (**F**).

**Figure 7.**
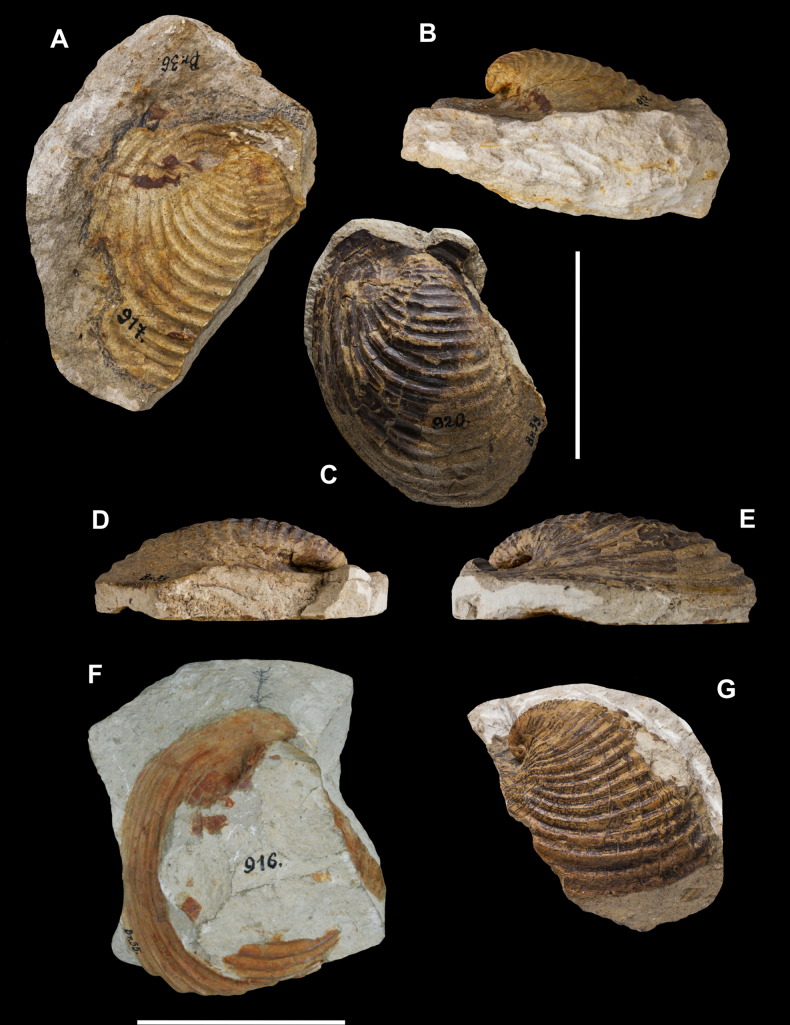
*Provalenciennesia limnaeoidea* (Gorjanović-Kramberger, 1901). **A, B**. Paralectotype specimen, Zagreb–Bačun (Borje), CNHM 5767-917, photo: Nives Borčić; **C–E**. Holotype of *Provalenciennesia poljaki* Gorjanović-Kramberger, 1923, Zagreb–Bačun (Borje), CNHM 5770-920, photo: Nives Borčić; **F**. Daruvar/Daruvár, CNHM 5766-916, photo: Nives Borčić; **G**. Lectotype specimen, Zagreb–Bačun (Borje), CNHM 5768-918, photo: Nives Borčić. Scale bars: 5 cm.

? 1856 *Limnaea Zelli* Hörn. – M. Hörnes, p. 606, pl. 49, fig. 23a–b. [syn. nov.?]
v *1890 Limnaea croatica Kramb.-Gorj. – Gorjanović-Kramberger, p. 154, pl. VI, figs 1–3.
*1904*Radix (Limnaea) croatica Kramberger-Gorjanović – Halaváts, p. 113.
*1923*Radix (Radix) croatica (Gorjanović-Kramberger) – Wenz, p. 1239. (cum syn.)
? *1923*Radix (Radix) zelli (Hörnes) – Wenz, p. 1314. (cum syn.)
v *1944*Radix (Limnaea) croatica Kr.-G. – Moos, p. 344.
1967 Radix croatica Gorjanović-Kramberger – Taktakishvili, text-fig. 7a.
v *1974*Radix (Limnaea) croatica (Gorjanović-Kramberger) – Milan et al., p. 81.
1975 Limnaea? cf. hyaloleuca Brusina, 1902 – Pană, p. 224, pl. IV, fig. 6.
1975 Radix balatonica Fuchs, 1870 – Pană, p. 225, pl. IV, fig. 7.
1985 Radix croatica (Gorj.-Kramb.) – Jámbor et al., pl. 23, fig. 4.
1987 Radix croatica (Gorj.-Kramb.) – Jámbor et al., pl. VIII, fig. 4.
? 1992 Radix sp. – Marinescu, fig. 3.
*1999*Radix (Radix) croatica – Vrsaljko, p. 22.
? v 2021 Radix cf. croatica (Gorjanović-Kramberger, 1890) – Botka et al., pp. 359–360, pl. I, figs 5–6.
v 2021 Radix croatica (Gorjanović-Kramberger, 1890) – Botka et al., pp. 359–360, pl. III, figs 17–19.

###### Type locality.

Moravče and Zagreb–Vrapče, Medvednica Mts., N Croatia (Gorjanović-Kramberger 1890).

###### Type material.

Syntypes: 3 specimens, CNHM 5178-343–5180-344 (Milan et al. 1974) and GS 1890/004/0001. In addition to the syntypes reposited in CNHM, a specimen collected by the author of the species is also available in the type collection of GS, subsequently labelled as “syntype”. Currently, two specimens of the original syntype material from Moravče are available in the CNHM and one specimen from Zagreb–Vrapče in the GS.

###### Material examined.

(65 specimens) ***Syntypes***. Croatia • 3 specs, syntypes; Moravče and Zagreb–Vrapče, Medvednica Mts., N Croatia; CNHM 5178-343 to 5180-344; GS 1890/004/0001.

###### Other material.

Croatia • 2 specs; Zagreb–Kostanjek/Podsused, Medvednica Mts., N Croatia; HNHM INV 2025.192. • 20 specs; Našice/Nekcse, Krndija Mts., N Croatia; HNHM INV 2025.205 to INV 2025.209, INV 2025.215, INV 2025.226, INV 2025.227. • 1 spec.; Voćin/Atyina, Papuk Mts., N Croatia; HNHM INV 2025.197. Romania • 8 specs; Sângeorgiu de Mureș/Marosszentgyörgy, Transylvanian Basin, Central Romania; SARA Pl.1543, Pl.1546, Pl.2024.635.1. • 2 specs; Valea Lungă/Hosszúaszó, Transylvanian Basin, Central Romania; SARA Pl.2024.420.1, Pl.2024.432.1. • 1 spec.; Micăsasa/Mikeszásza, Transylvanian Basin, Central Romania; SARA Pl.2024.322.1. • 6 specs; Lopadea Veche/Oláhlapád, Transylvanian Basin, Central Romania; HNHM INV 2025.230, INV 2025.247, INV 2025.249. • 2 specs; Sighișoara/Segesvár/Schäßburg, Transylvanian Basin, Central Romania; HNHM INV 2025.256, INV 2025.257. • 1 spec.; Viforoasa/Havadtő, Transylvanian Basin, Central Romania; HNHM INV 2025.253. Hungary • 18 specs; Pécs-Danitzpuszta, Mecsek Mts., S Hungary; HNHM INV 2025.187 to INV 2025.189. • 1 spec.; Hidas-53 borehole, Mecsek Mts., S Hungary; HNHM INV 2025.186.

###### Note.

For collectors and specimen-related information see Suppl. material 1.

###### Description.

Shell thin, smooth, subglobose or oviform with fine, sometimes strong growth lines. Dextral shell coiling and three curved whorls. A strongly widened body whorl with an oval and rimmed aperture. A small depression can be observed on upper part of aperture. Apertural edge sharp, columellar lip bent inwards. Protoconch could be observed only in well-preserved specimens, it is simple and smooth. Size parameters: L = 10–23 mm, W = 5.5–15.5 mm, L/W = 0.9–1.9, LBw = 6.5–19 mm.

###### Distribution and stratigraphic range.

This species was described and mentioned from old Pannonian offshore (sublittoral and profundal) marls all over the Pannonian Basin: Moravče and Zagreb–Vrapče (Gorjanović-Kramberger 1890) and Zagreb–Kostanjek/Podsused, Medvednica Mts., N Croatia (Vrsaljko 1999; this study); Gojlo-4 and Bujavica-9 boreholes, Sava Basin, N Croatia (Moos 1944); Lajoskomárom-1 borehole, Central Hungary (Jámbor et al. 1985, 1987); Tata, Gerecse Mts., N Hungary (Cziczer et al. 2009); Bisericani/Székelyszentlélek and Bulgăreni/Bogárfalva, Transylvanian Basin, Central Romania (Pană 1975); B-1 borehole in Belgrade, Šumadija Hills, Central Serbia (Rundić et al. 2011); Lopadea Veche/Oláhlapád, Sighișoara/Segesvár/Schäßburg, Viforoasa/Havadtő, Sângeorgiu de Mureș/Marosszentgyörgy, Valea Lungă/Hosszúaszó, Micăsasa/Mikeszásza, Transylvanian Basin, Central Romania; Hidas-53 borehole, Mecsek Mts., S Hungary; Našice/Nekcse, Krndija Mts., Voćin/Atyina, Papuk Mts., N Croatia (this study). It is also recovered from the uppermost Sarmatian layers recently from Pécs-Danitzpuszta, Mecsek Mts., S Hungary (Botka et al. 2021) and a questionable specimen (described as “*Limnaea Zelli*”) was found in the vicinity of Valtice/Feldsberg, Vienna Basin, Czech Republic (M. Hörnes 1856). It is a common member of the “*Lymnocardium*” *praeponticum* – *Radix croatica* dwarf mollusc fauna. “*Lymnocardium*” *praeponticum* – *Radix croatica* mollusc biozone = “Croatica beds” or “Croatica Formation” in Croatia (ca 11.6–11.4 Ma).

###### Remarks.

Botka et al. (2021) raised the possibility that *Lymnaea extensa* Gorjanović-Kramberger, 1890 is a steinkern of *Radix croatica* specimens. Subsequent investigations showed, however, that *L. extensa* does not show the internal structure and ornamentation of *R. croatica*. In fact, it can be rather assigned into the genus *Stagnicola* based on its four whorls and elongated structure. *Lymnaea zelli* described by M. Hörnes (1856), however, is probably a senior synonym of *R. croatica*. The only one recovered and figured specimen and uncertain position of the locality make this statement questionable. The holotype was not found in the collection of the NHMV and thus it is presumed lost. Even if we accept *L. zelli* (nomen oblitum) as a senior synonym of *R. croatica*, we recommend keeping the name *R. croatica* (nomen protectum) in use according to the Article 23.9.2 of ICZN (1999), because this species has been referred in the literature as *R. croatica* since 1890. Informal and formal lithostratigraphic units commonly used in Croatia (“Croatica beds” or “Croatica Formation”) are also connected to this name (Vrsaljko 1999; Sebe et al. 2020; Anđelković and Radivojević 2021). Limnaea? cf. hyaloleuca and *Radix balatonica* mentioned by Pană (1975) are probably misidentifications due to poor preservation of the studied material. Marinescu (1992) mentioned a common lymnaeid species from the Sarmatian of the Borod Basin as *Radix* sp. There is no description of the given species; however, the figured specimen bears the characteristics of *R. croatica*.

##### Genus Velutinopsis

Taxon classificationAnimaliaLymnaeidaLymnaeidae

Sandberger, 1875

EA9D012C-54CB-5E02-955E-BD361F70F688

###### Type species.

*Velutinopsis velutina* (Deshayes, 1838); original designation.

###### Original description.

“Shell is egg-shaped, very strongly globose, smooth, in the middle part slightly sunken, on the basis strongly curved, and with narrow and in later age fully closed umbo. It consists of three bulges, in the outer side rapidly increasing in width and therefore wide and deep channel-like sutures dividing whorls ornamented by obliquely situated growth lines. The last and largest whorl is ~ 5× larger than the previous one. The aperture is widely egg-shaped, oblique, open, and bears an obtuse rim” (in German in Sandberger 1875, translated by D. Botka). [Originally, it was described as a subgenus. Wenz (1923) and Moos (1944) were the first to raise it to the genus level. Gorjanović-Kramberger (1923) classified *Lymnaea velutina*, *L. rugosa*, and *L. nobilis* into this genus].

###### Emended diagnosis.

Shell thin, smooth, convex, and globose with dense and fine or thickened growth lines or furrows. Dextral shell coiling, three curved whorls rapidly increasing in width. A very strongly widened inflated body whorl with an oval and rimmed aperture. Whorls divided by wide and deep, channel-like sutures. Body whorl fully covers previous whorl and protoconch of spire, Bw is ~ 8× larger than previous whorl. Apertural edge sharp. Umbo closed in adult specimens. Protoconch could be observed only in well-preserved or in laterally compacted specimens, it is simple and smooth.

##### Velutinopsis velutina

Taxon classificationAnimaliaLymnaeidaLymnaeidae

(Deshayes, 1838)

F65FC616-402A-5A4F-AC65-816892E35B82

**Figure 8.**
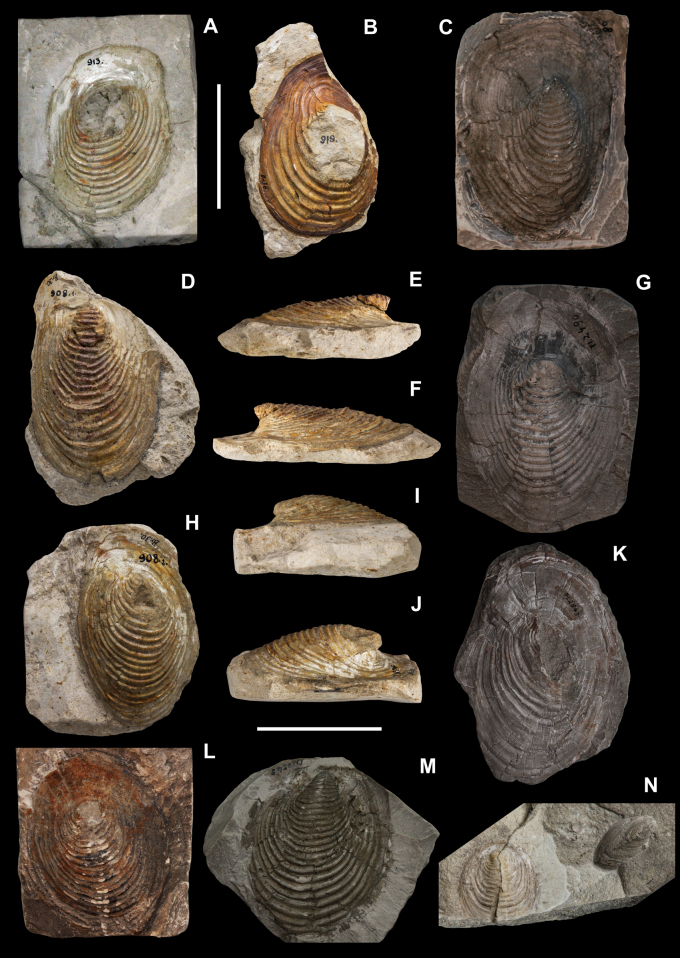
*Provalenciennesia arthaberi* (Gorjanović-Kramberger, 1901). **A**. Lectotype specimen, Zagreb–Podsljeme (Gračani), CNHM 5763-913, photo: Nives Borčić; **B**. Holotype specimen of *Provalenciennesia langhofferi* (Gorjanović-Kramberger, 1901), Zagreb–Bačun (Borje), CNHM 5769-919, photo: Nives Borčić; **C**. Holotype specimen of *Provalenciennesia schafarziki* (Gorjanović-Kramberger, 1901), Beočin/Beocsin/Belcsény, SARA Pl.2489; **D–F**. Zagreb–Podsljeme (Gračani), CNHM 5758-908/1, photo: Nives Borčić; **G**. Beočin/Beocsin/Belcsény, SARA Pl.2490; **H–J**. Zagreb–Podsljeme (Gračani), CNHM 5758-908/2, photo: Nives Borčić; **K**. Beočin/Beocsin/Belcsény, SARA Pl.2543; **L**. Beočin/Beocsin/Belcsény, SARA Pl.6667; **M**. Beočin/Beocsin/Belcsény, SARA Pl.2545; **N**. Juvenile specimen with a *Congeria banatica*–*Carinatocongeria digitifera* transitional form, Zagreb–Vugrovec, CNHM 1161, photo: Nives Borčić. Scale bars: 5 cm.

**Figure 9.**
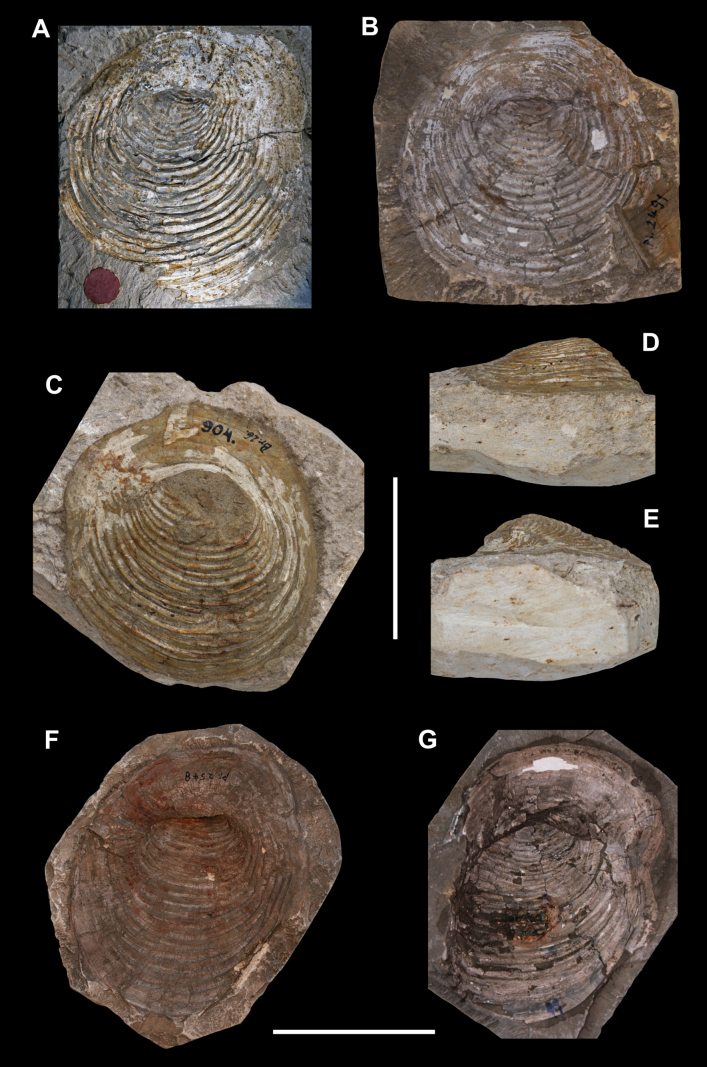
*Provalenciennesia pauli* (R. Hörnes, 1875). **A**. Holotype specimen, Gornji Kneginec, GS 1875/006/0022, photo: Lajos Katona; **B**. Beočin/Beocsin/Belcsény, SARA Pl.2491; **C–E**. Zagreb–Podsljeme (Gračani), CNHM 5754-904, photo: Nives Borčić; **F**. Beočin/Beocsin/Belcsény, SARA Pl.2548; **G**. Tata, SARA Pl.4771. Scale bars: 5 cm.

**Figure 10.**
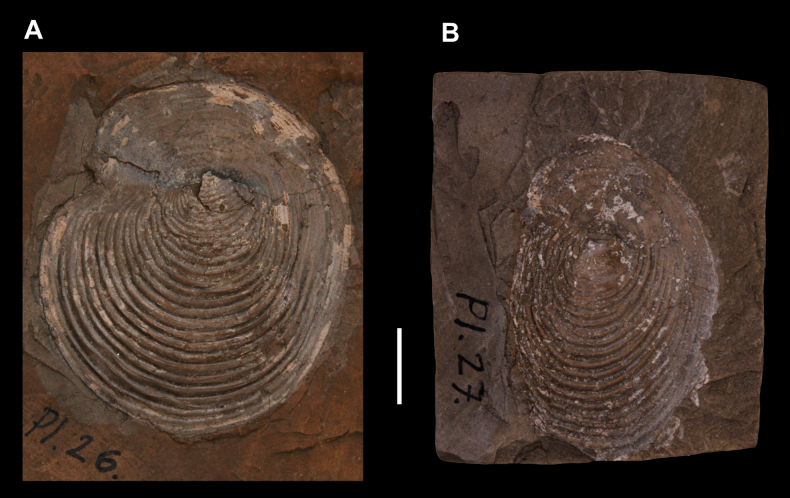
*Provalenciennesia boeckhi* (Halaváts, 1886). **A**. Lectotype specimen, Ciuchici/Csukics/Tyukó, SARA Pl.26; **B**. Paralectotype specimen, Ciuchici/Csukics/Tyukó, SARA Pl.27. Scale bar: 1 cm.

**Figure 11.**
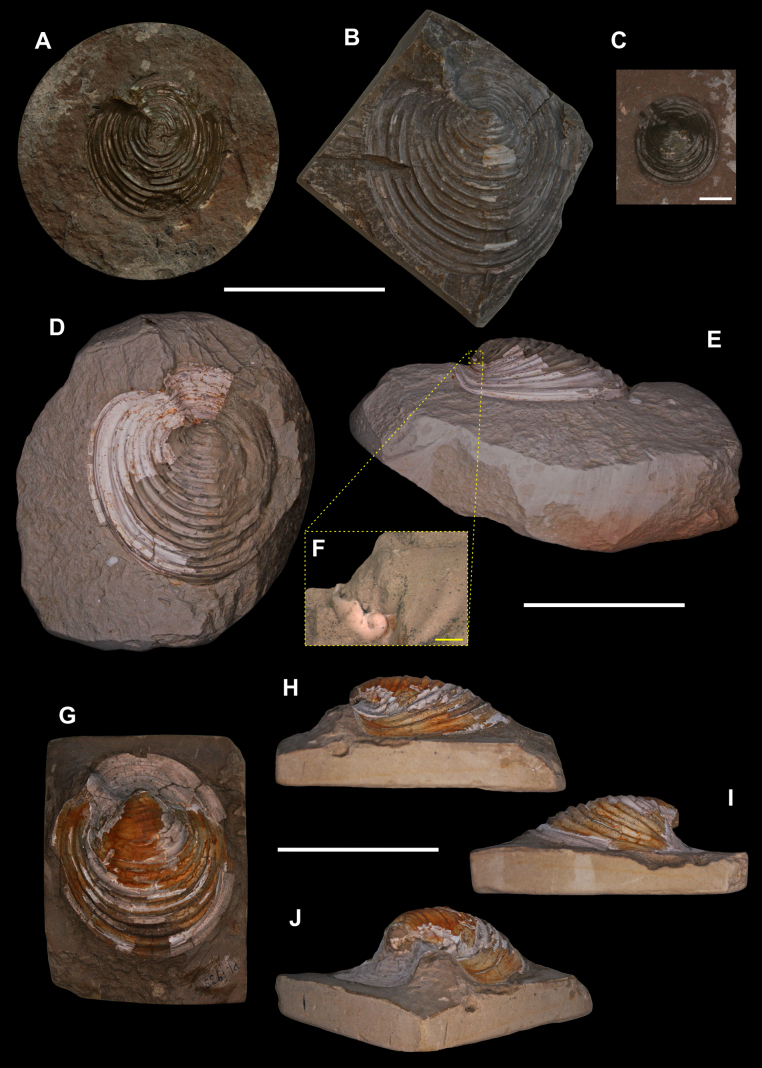
*Valenciennius reussi* Neumayr, 1875. **A**. Algyő-16 borehole, SARA Pl.2024.67.1; **B**. Szekszárd, SARA Pl.3029; **C**. juvenile specimen, Algyő-160 borehole, SARA Pl.2024.83.1; **D–F**. Bátaszék, HNHM PAL 2025.10.1; **G–J**. Tirol/Királykegye, SARA Pl.1939. Scale bars: 5 cm (white), 1 cm (black), 1 mm (yellow).

**Figure 12.**
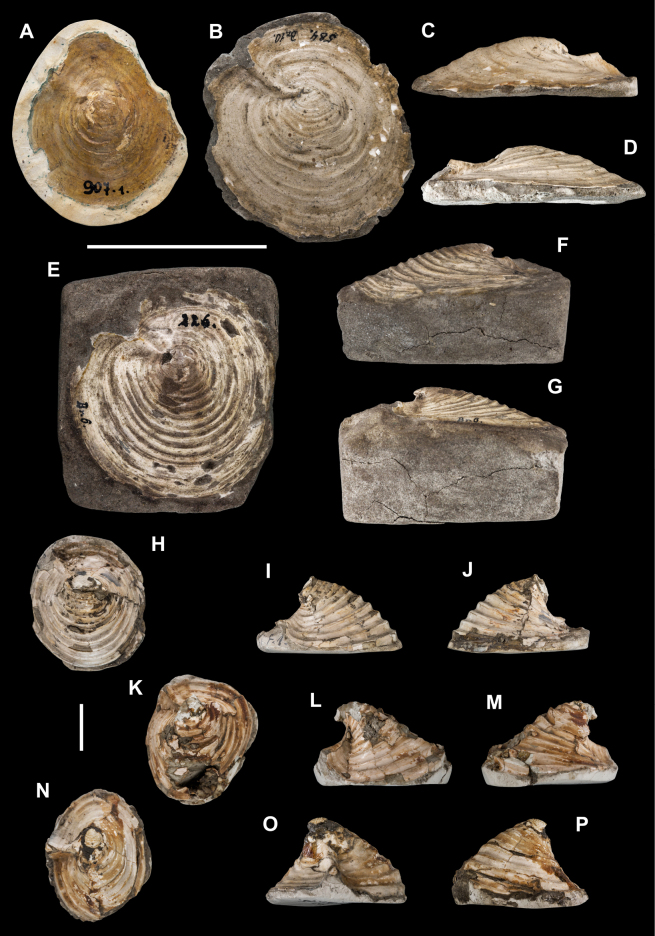
**A**. *Provalenciennesia* sp. indet. juvenile holotype specimen of *Provalenciennesia pauli intermedia* (Gorjanović-Kramberger, 1901), *Provalenciennesia pauli*–*Valenciennius reussi* transitional form, Zagreb–Šestine, CNHM 5757-907/1, photo: Nives Borčić; **B–P**. *Valenciennius reussi* Neumayr, 1875; **B–D**. Neotype specimen of *Valenciennius peltus* Brusina, 1878, Zagreb–Okrugljak, CNHM 5064-229, photo: Nives Borčić; **E–G**. Holotype specimen of *Valenciennius brusinai* Gorjanović-Kramberger, 1901, Zagreb–Okrugljak, CNHM 5061-226, photo: Nives Borčić; **H–J**. Juvenile syntype specimen of *Valenciennius altus* Gorjanović-Kramberger, 1901, Zagreb–Okrugljak, CNHM 5066-231/1, photo: Nives Borčić; **K–M**. Juvenile syntype specimen of *Valenciennius altus* Gorjanović-Kramberger, 1901, Zagreb–Okrugljak, CNHM 5066-231/2, photo: Nives Borčić; **N–P**. Juvenile syntype specimen of *Valenciennius altus* Gorjanović-Kramberger, 1901, Zagreb–Okrugljak, CNHM 5066-231/3, photo: Nives Borčić. Scale bars: 5 cm (**A–G**), 1 cm (**H–P**).

**Figure 13.**
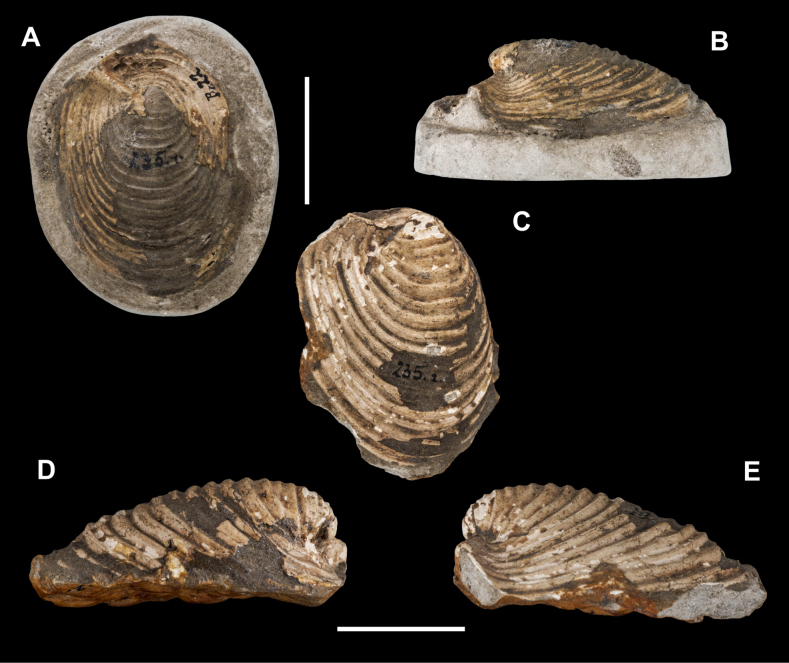
**A, B**. *Valenciennius kiseljaki* Gorjanović-Kramberger, 1901, lectotype specimen, Zagreb–Okrugljak, CNHM 5070-235/1, photo: Nives Borčić; **C–E**. *Valenciennius cf. kiseljaki*, paralectotype specimen, Zagreb–Okrugljak, CNHM 5070-235/2, photo: Nives Borčić. Scale bars: 2 cm.

**Figure 14.**
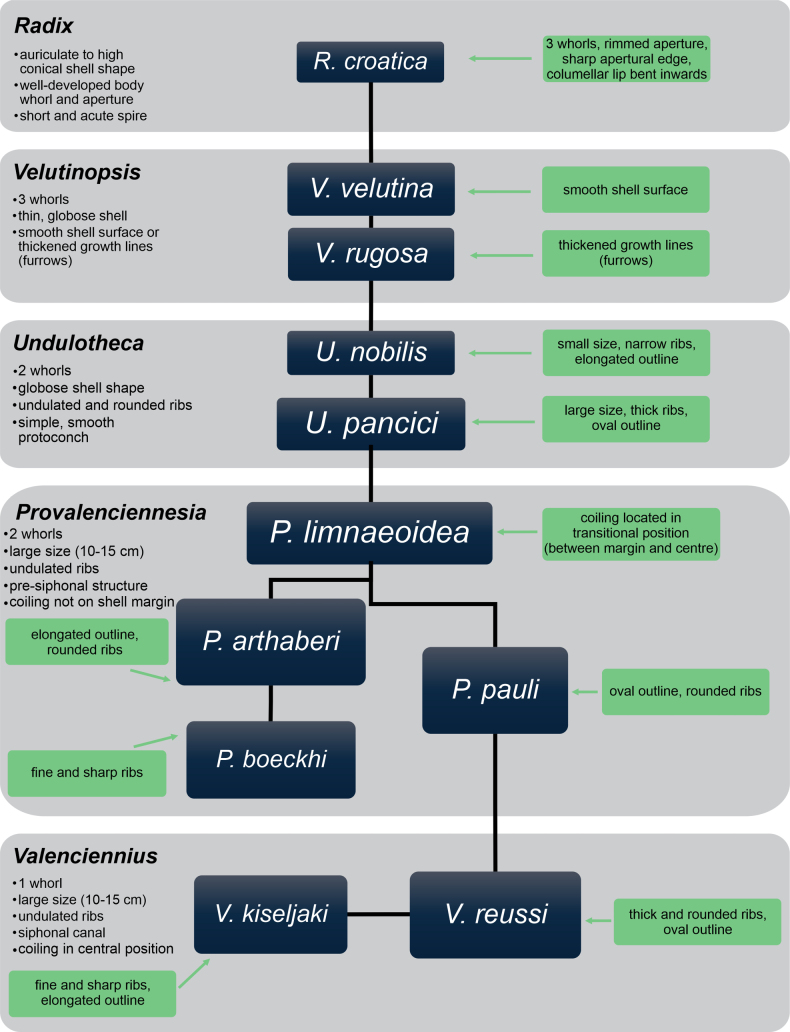
An identification key for the deep-water lymnaeid molluscs of Lake Pannon. Genus-level morphological characters are indicated on the left side, while species-related characters are in the green fields. Black lines represent suggested phylogenetic relationships, while the sizes of the dark blue fields relates to average shell size.

**Figure 15.**
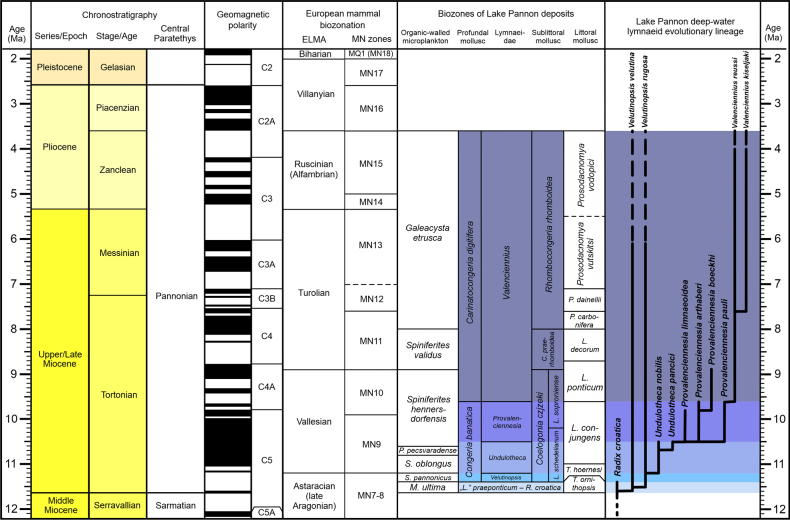
Chronostratigraphic chart of the Pannonian Stage (edited with TimeScale Creator 8.1) with the European mammal biozones, Lake Pannon biozones, and Lake Pannon deep-water lymnaeid evolutionary lineage. Different blue shades indicate deep-water (sublittoral to profundal) biozones based on lymnaeid evolution as follows: 11.6–11.4 Ma – “*Lymnocardium*” *praeponticum* – *Radix croatica* zone, 11.4–11.2 Ma – *Velutinopsis* zone, 11.2–10.5 Ma – *Undulotheca* zone, 10.5–9.6 Ma – *Provalenciennesia* zone, and 9.6–3.6 Ma – *Valenciennius* zone. Organic-walled microplankton: M: *Mecsekia*, S: *Spiniferites*, P: *Pontiadinium*. Molluscs: L: *Lymnocardium*, R: *Radix*, T: *Trigonipraxis*, C: *Congeria*, P: *Prosodacnomya*. Source: modified from Hilgen et al. (2012), Magyar and Geary (2012), and Magyar et al. (2025).

**Figure 16.**
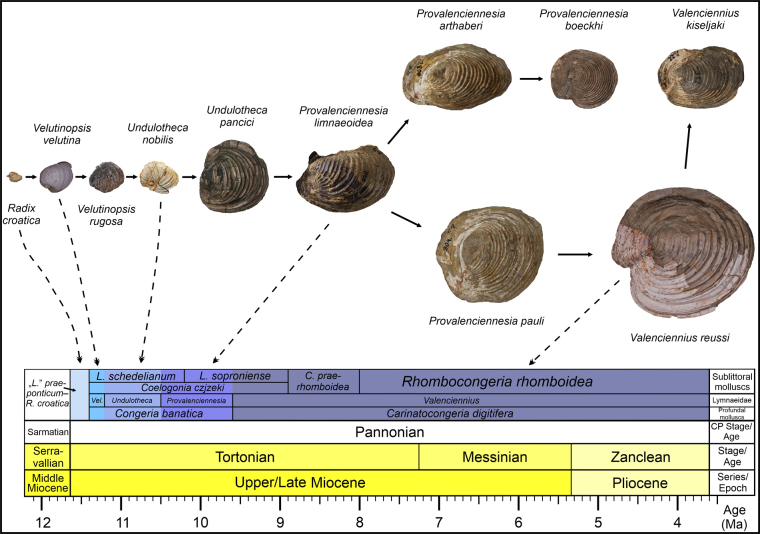
Evolutionary lineage of the Lake Pannon deep-water lymnaeid gastropods with a chronostratigraphic chart of the Pannonian Stage and with the Lake Pannon sublittoral and profundal mollusc biozones. Characteristic mollusc specimens are to scale. Different blue shades indicate deep-water (sublittoral to profundal) biozones based on lymnaeid evolution as follows: 11.6–11.4 Ma – “*Lymnocardium*” *praeponticum* – *Radix croatica* zone, 11.4–11.2 Ma – *Velutinopsis* zone, 11.2–10.5 Ma – *Undulotheca* zone, 10.5–9.6 Ma – *Provalenciennesia* zone, and 3.6–9.6 Ma – *Valenciennius* zone. Molluscs: L: *Lymnocardium*, R: *Radix*, C: *Congeria*, Vel: *Velutinopsis*.

Figs 2, 3 A–M

v *1838 Limnaea velutina – Deshayes, p. 64, pl. V, figs 12–14.
1842 Limnea velutina Desh. – Rousseau, p. 790, pl. 3, fig. 2–2b.
1855 Limnaea velutina Deshayes – Bourguignat, p. 83, pl. 5, figs 2–3.
v p 1868 Limnaeus nobilis n. sp. – Reuss, pp. 85–87.
1875 Limneus (Velutinopsis) velutinus Deshayes – Sandberger, pp. 700–701, pl. XXXII, figs 10–10a.
v 1899 Limnaeus velutinus Desh. – Gorjanović-Kramberger, p. 126, pl. V, fig. 1.
v 1899 Limnaeus simplex Kramb.-Gorj. – Gorjanović-Kramberger, p. 126, pl. V, fig. 3. [new synonym]
v 1901 Limnaea velutina Desh. – Gorjanović-Kramberger, p. 137, pl. X, fig. 15.
*1904 Limnaeus simplex* Kramberger-Gorjanović – Halaváts, p. 114.
v *1904 Limnaeus velutinus* Deshayes – Halaváts, p. 115.
*1923 Velutinopsis simplex* (Gorjanović-Kramberger) – Wenz, p. 1325. (cum syn.)
*1923 Velutinopsis velutina* (Deshayes) – Wenz, pp. 1326–1327. (cum syn.)
1928 Limnaea (Velutinopsis) spec. (aff. velutina Desh. sp.) – Pavlović, p. 36.
1942 Radix (Velutinopsis) cf. velutina (Deshayes) – Wenz, p. 68, pl. 24, fig. 380.
v 1944 Radix (Limnaea) simplex Kr.-G. – Moos, p. 344.
v 1944 Radix kobelti Brus. – Moos, pp. 344–345, pl. XXI, figs 1–2.
v 1944 Velutinopsis velutina Desh. – Moos, pp. 345–347, pl. XXI, figs 3–5.
1951 Lymnaea kobelti Brus. – Stevanović, pl. XII, fig. 8a–b.
1956 Radix kobelti Brusina – Papp, p. 71.
1959 Radix (Velutinopsis) velutina (Deshayes) – Wenz, p. 95, text-fig. 307.
1967 Velutinopsis velutina Rousseau [sic!] – Taktakishvili, text-fig. 7b.
1969 Velutinopsis velutina (Deshayes, 1838) – Marinescu, pp. 314–315, pl. I, figs 4–5b.
1969 Velutinellus pilleus g. n., sp. n. – Marinescu, pp. 319–320, text-figs 4A, 5, pl. I, figs 6a–8b. [syn. nov.?]
1971 Lymnaea (Radix) velutinaDeshayes 1838 – Széles in Góczán and Benkő, p. 338.
1973 Radix paucispira (Fuchs, 1870) – Marinescu, p. 42, pl. XII, fig. 2.
v *1974*Radix (Lymnaea) simplex (Kramberger-Gorjanović) – Milan et al., p. 83.
1975 Radix paucispira (Fuchs, 1870) – Pană, p. 226, pl. IV, figs 8–11.
1977 Velutinopsis velutina (Desh.) – Lubenescu and Lubenescu, pl. II, figs 9–10.
1981 Velutinopsis velutina (Deshayes, 1838) – Lubenescu, p. 181, pl. XIV, fig. 4.
1983 Velutinopsis kobelti (Brus.) – Korpás-Hódi, pl. X, fig. 1.
1985 Radix kobelti Reuss [sic!] – Stevanović and Papp, pl. 27, fig. 11.
1992 Velutinopsis cf. velutina (Desh.) – Korpás-Hódi, pl. II, fig. 3.
v 1999 Radix (Velutinopsis) velutina (Deshayes) – Lennert et al., pl. V, fig. 5.
2009 Radix kobelti (Brusina 1884) – Cziczer et al., fig. 6: n.
2013 Radix kobelti Reuss [sic!] – ter Borgh et al., fig. 9: 19.
2013 Radix kobelti (Brusina, 1884) – Katona et al., fig. 4: a.
2016 Velutinopsis velutina – Lubenescu, pl. I, fig. 16(p). [cop. Marinescu 1969]
v 2019 Velutinopsis velutina (Deshayes, 1838) – Botka et al., fig. 3: l.
? v 2021 "*Lymnocardium*” *margaritaceum* – Botka et al., pl. IV, fig. 11.
v 2021 Velutinopsis sp. – Botka et al., pl. IV, fig. 12.
v 2023 Velutinopsis velutina (Deshayes, 1838) – Neubauer, fig. 6.7: e.

###### Type locality.

Kerch–Kamysh-Burun, Black Sea Basin, Ukraine/Russia (Deshayes 1838).

###### Type material.

Holotype (by monotypy): UL UCBL-EM 33436.

###### Material examined.

(176 specimens) ***Holotype***. Ukraine/Russia • 1 spec., holotype; Kerch–Kamysh-Burun, Black Sea Basin; UL UCBL-EM 33436. Croatia • 1 spec., holotype (by monotypy) of *Velutinopsis simplex*; Londžica/Babindol, Krndija Mts., N Croatia; CNHM 5449-599. ***Paralectotypes***. Romania • 2 specs, paralectotypes of *Undulotheca nobilis*; Şura Mare/Nagycsűr–Hamba/Kakasfalva/Hahnbach, Transylvanian Basin, Central Romania; GS 1868/005/0001.

###### Other material.

Croatia • 1 spec.; Zagreb–Čučerje, Medvednica Mts., N Croatia; CNHM 984b. • 9 specs; boreholes: Peklenica-47, Mura Basin (2 specs), Grubišno Polje-1, Bednja-2, Osekovo-1, Janja Lipa-1, Gojlo-4, Sava Basin, Ludbreg-1, Drava Basin, Mur-1, Mura Basin, N Croatia; CNHM 1 to 9. • 1 spec.; Zagreb, exact locality unknown, Medvednica Mts., N Croatia; SARA Pl.2482. • 3 specs; Mirti/Hruševec, Zagorje Basin, NW Croatia; HNHM INV 2025.194, INV 2025.195. • 18 specs; Našice/Nekcse, Krndija Mts., N Croatia; HNHM INV 2025.198 to INV 2025.203, INV 2025.210, INV 2025.211, INV 2025.214, INV 2025.217, INV 2025.220, INV 2025.223. Romania • 4 specs; Gârbovița/Középorbó, Transylvanian Basin, Central Romania; SARA Pl.2024.561.1, Pl.2024.562.1, Pl.2024.563.1, Pl.2024.564.1. • 2 specs; Sântimbru/Marosszentimre, Transylvanian Basin, Central Romania; SARA Pl.2024.445.1, Pl.2024.446.1. • 2 specs; Miercurea Sibiului/Szerdahely, Transylvanian Basin, Central Romania; SARA Pl.2024.143.1, Pl.2024.144.1. • 1 spec.; Glogoveț/Kisgalgóc, Transylvanian Basin, Central Romania; SARA Pl.2024.398.1. • 2 juvenile specs; Râmeț/Remete, Transylvanian Basin, Central Romania; SARA Pl.1817. • 7 specs; Axente Sever/Asszonyfalva, Transylvanian Basin, Central Romania; SARA Pl.2024.200.1, Pl.2024.204.1, Pl.2024.222.1, Pl.2024.227.1, Pl.2024.228.1, Pl.2024.229.1, Pl.2024.230.1. • 3 specs; Sângeorgiu de Mureș/Marosszentgyörgy, Transylvanian Basin, Central Romania; SARA Pl.1544 to Pl.1545. • 1 spec.; Corund/Korond, Transylvanian Basin, Central Romania; BBU 2127. • 1 spec.; Bodogaia/Alsóboldogfalva, Transylvanian Basin, Central Romania; NGM-GIR P-1506. • 8 specs; Gușterița/Szenterzsébet/Hammersdorf, Transylvanian Basin, Central Romania; BM48.917, 48.919, 48.921 to 48.928, 48.932, 48.933 to 48.935, 48.936 to 48.938. • 1 spec.; Sângeorgiu de Mureș/Marosszentgyörgy, Transylvanian Basin, Central Romania; MIM 015. • 1 spec.; Odorheiu Secuiesc/Székelyudvarhely, Transylvanian Basin, Central Romania; MIM 803. • 2 specs; Zorlențu Mare/Alsózorlenc, Banat Mts., W Romania; BBU 21346. • 2 specs; Câmpia/Néramező/Langenfeld, Almăjului Mts., SW Romania; SARA Pl.1878. • 1 spec.; Chelința/Kelence, Șimleu Basin, NW Romania; SARA Pl.2212. • 1 spec.; Bodogaia/Alsóboldogfalva, Transylvanian Basin, Central Romania; NGM-GIR P-1506. • 1 spec.; Gârbovița/Középorbó, Transylvanian Basin, Central Romania; HNHM INV 2025.229. • 3 juvenile specs; Mihalț/Mihálcfalva, Transylvanian Basin, Central Romania; HNHM INV 2025.234. • 5 specs; Lopadea Veche/Oláhlapád, Transylvanian Basin, Central Romania; HNHM INV 2025.246, INV 2025.248, INV 2025.250. • 1 juvenile spec.; Sighișoara/Segesvár/Schäßburg, Transylvanian Basin, Central Romania; HNHM INV 2025.258. • 7 specs; Tău/Székástóhát, Transylvanian Basin, Central Romania; HNHM INV 2025.232. • 8 specs; Gușterița/Szenterzsébet/Hammersdorf, Transylvanian Basin, Central Romania; HNHM INV 2025.237, INV 2025.245, INV 2025.252. SERBIA • 14 specs; Beočin/Beocsin/Belcsény, Fruška Gora, N Serbia; SARA Pl.2543, Pl.2492 to Pl.2495. • 2 specs; Nikolinci/Nicolinţ/Temesmiklós, Zagajica Basin, NE Serbia; SARA Pl.1921. • 6 specs; Belgrade–Umka, Šumadija Hills, Central Serbia; NHMB 1862. Bosnia and Herzegovina • 4 specs; Tuzla–Kreka, Tuzla Basin, NE Bosnia and Herzegovina; NHMB 2076. Hungary • 2 specs; Sümeg, Bakony Mts., W Hungary; SARA Pl.4868, Pl.4870. • 39 specs, MOL drillcore collection; boreholes: Vése-4 (SARA Pl.2024.41.1, Pl.2024.43.1), Liszó-4 (SARA Pl.2024.25.1, Pl.2024.26.1, Pl.2024.27.1), Bajcsa-26, Bajcsa-29, Bajcsa-35, Belezna-12, Belezna-20, Belezna-21, Liszó-2, Mesztegnyő-1, Őriszentpéter-1, Semjénháza-1, Semjénháza-2, Szenta-2, Táska-4 (SARA Pl.2024.5.1, Pl.2024.6.1, Pl.2024.7.1, Pl.2024.11.1, Pl.2024.12.1, Pl.2024.14.1, Pl.2024.24.1, Pl.2024.28.1, Pl.2024.35.1, Pl.2024.36.1, Pl.2024.37.1, Pl.2024.38.1, Pl.2024.39.1), Zala Basin, W Hungary, Ölbő-4, Danube Basin, NW Hungary (SARA Pl.2024.34.1), Szank-1, Tázlár Basin (SARA Pl.2024.55.1, Pl.2024.56.1, Pl.2024.57.1), Algyő-34 (SARA Pl.2024.73.1), Algyő-38 (SARA Pl.2024.74.1), Algyő-39 (SARA Pl.2024.75.1, Pl.2024.76.1), Algyő-94 (SARA Pl.2024.81.1), Ferencszállás-11, Algyő High (SARA Pl.2024.93.1), Tótkomlós-21, Békés Basin (SARA Pl.2024.109.1, Pl.2024.110.1), Kiskundorozsma-4 (SARA Pl.2024.48.1), Öttömös-1, Szeged Basin (SARA Pl.2024.50.1), Pusztaföldvár-204, Battonya-Pusztaföldvár High, S Hungary (SARA Pl.2024.104.1), Fábiánsebestyén-3, Fábiánsebestyén Basin, SE Hungary (SARA Pl.2024.91.1, Pl.2024.92.1), Tóalmás-3 (SARA Pl.2024.60.1), Nagykörű-3, Jászság Basin, Central Hungary (SARA Pl.2024.100.1). • 1 spec.; Pécs–Nagyárpád, Mecsek Mts., S Hungary; HNHM INV 2025.173. • 7 specs; Bátaszék, Szekszárd Hills, S Hungary; HNHM INV 2025.174 to INV 2025.178, INV 2025.179, INV 2025.180.

###### Note.

For collectors and specimen-related information see Suppl. material 1.

###### Description.

Shell thin, smooth, convex, and globose with dense and fine growth lines. Dextral shell coiling, three curved whorls rapidly increasing in width and a very strongly widened inflated body whorl with an oval and rimmed aperture. Whorls divided by wide and deep, channel-like sutures. Body whorl fully covers previous whorl and protoconch of spire, it is ~ 8× larger than previous whorl. Apertural edge sharp. Umbo closed in adult specimens. Protoconch could be observed only in well-preserved or in laterally compacted specimens, it is simple and smooth. Size parameters: L = 9–41 mm, W = 8–35.5 mm, L/W = 0.7–1.4.

###### Distribution and stratigraphic range.

This species was described and mentioned from Pannonian offshore (sublittoral and profundal) marls all over the Pannonian Basin: Londžica/Babindol (Gorjanović-Kramberger 1899) and Našice/Nekcse, Krndija Mts. (this study), Zagreb-Lukšić (Gorjanović-Kramberger 1923), Zagreb–Čučerje (Kochansky-Devidé and Pikija 1976), and Zagreb–Kostanjek/Podsused, Medvednica Mts., N Croatia (Papp 1956; this study); Mirti/Hruševec (this study) and Novi Marof, Zagorje Basin, NW Croatia (Gorjanović-Kramberger 1901); Peklenica-47 and Mur-1 boreholes, Mura Basin, Grubišno Polje-1, Bednja-2, Osekovo-1, Janja Lipa-1, Gojlo-4 boreholes, Sava Basin, and Ludbreg-1 borehole, Drava Basin, N Croatia (Moos 1944; Sokač 1990); Şura Mare/Nagycsűr–Hamba/Kakasfalva/Hahnbach (Reuss 1868), Gârbovița/Középorbó (this study), Sântimbru/Marosszentimre (Telegdi-Roth 1903; this study), Glogoveț/Kisgalgóc (Telegdi-Roth 1907; this study), Axente Sever/Asszonyfalva (Telegdi-Roth 1909; this study), Sângeorgiu de Mureș/Marosszentgyörgy (Jaskó 1950; this study), Archita/Erked (Pană 1975), Miercurea Sibiului/Szerdahely (Halaváts 1907; Lubenescu and Lubenescu 1977; Lubenescu 1981), Vingard/Vingárd (Lubenescu and Lubenescu 1977; Lubenescu 1981), Gârbova/Szászorbó (Lubenescu and Lubenescu 1977; Lubenescu 1981), Valea Rodului (Lubenescu and Lubenescu 1977; Lubenescu 1981), Săcădate/Oltszakadát (Lubenescu and Lubenescu 1977; Lubenescu 1981), Gușterița/Szenterzsébet/Hammersdorf (Lubenescu and Lubenescu 1977; Lubenescu 1981; Botka et al. 2019; this study), Corund/Korond, Bodogaia/Alsóboldogfalva, Odorheiu Secuiesc/Székelyudvarhely, Mihalț/Mihálcfalva, Lopadea Veche/Oláhlapád, Râmeț/Remete, Sighișoara/Segesvár/Schäßburg, Tău/Székástóhát, Transylvanian Basin, Central Romania (this study); Zorlențu Mare/Alsózorlenc, Banat Mts., W Romania (Marinescu et al. 1977; this study); Câmpia/Néramező/Langenfeld, Almăjului Mts. (Halaváts 1882), Rădmănești/Radmanóc, Apuseni Mts., W Romania (Marinescu 1973); Chelința/Kelence, Șimleu Basin, NW Romania (this study); Beočin/Beocsin/Belcsény, Fruška Gora, N Serbia (Koch 1902; Halaváts 1904; Stevanović and Papp 1985; ter Borgh et al. 2013); Nikolinci/Nicolinţ/Temesmiklós, Zagajica Basin, NE Serbia (Marinescu et al. 1977; this study); Jazovnik, Obrenovac Graben, W Serbia (Stevanović 1990b); Belgrade–Umka and Ripanj, Šumadija Hills, Central Serbia (Pavlović 1928; this study); Tuzla–Kreka, Tuzla Basin, NE Bosnia and Herzegovina (this study); Sümeg, Bakony Mts., W Hungary (this study); Szombathely-II, Ölbő-4, and Mocsa Mct-2 boreholes, Danube Basin, NW Hungary (Korpás-Hódi 1983, 1992; this study); Tata, Gerecse Mts., N Hungary, Kisbér and Devecser, Bakony Mts., W Hungary, and Bátaszék, Szekszárd Hills, S Hungary (Lennert et al. 1999; Cziczer et al. 2009; this study); Bajcsa-26, -29, -35, Belezna-12, -20, -21, Liszó-2, -4, Mesztegnyő-1, Őriszentpéter-1, Semjénháza-1, -2, Szenta-2, Táska-4, and Vése-4 boreholes, Zala Basin, W Hungary (this study); Szank-1 borehole, Tázlár Basin, Algyő-34, Algyő-38, -39, -94, and Ferencszállás-11 boreholes, Algyő High, Tótkomlós-21 borehole, Békés Basin, Kiskundorozsma-4 and Öttömös-1 boreholes, Szeged Basin, Pusztaföldvár-204 borehole, Battonya-Pusztaföldvár High, S Hungary (this study); Fábiánsebestyén-3 borehole, Fábiánsebestyén Basin, SE Hungary (this study); Tóalmás-3 and Nagykörű-3 boreholes, Jászság Basin, Central Hungary (this study); Kozármisleny (Katona et al. 2013); Pécs–Nagyárpád and Pécs-Danitzpuszta, Mecsek Mts., S Hungary (Szónoky et al. 1999; Botka et al. 2021; this study). There are some reports from outside the Pannonian Basin, namely from the area of the Eastern Paratethys from the upper Maeotian and Pontian deposits (Kerch–Kamysh-Burun, Black Sea Basin, Ukraine/Russia – Deshayes 1838; Rousseau 1842; Wenz 1959; Apostolache – Wenz 1942, Bengești and Crăguești, Dacian Basin, S Romania – Marinescu 1969). The species is widespread throughout the entire Pannonian Stage. It is a rare member of the “*L.*” *praeponticum* – *R. croatica* mollusc biozone and a common member of the *Congeria banatica* and *Carinatocongeria digitifera* mollusc biozones (ca 11.6–3.6 Ma). Based on the FAD of *V. velutina* and co-occurrence with *R. croatica* and *V. rugosa*, a *Velutinopsis* lineage zone is proposed (ca 11.4–11.2 Ma). This species is originated from Lake Pannon, but it successfully migrated into the Eastern Paratethys region in the late Maeotian. It is reported from the *Paradacna*- and *Valenciennius*-bearing deep-water upper Maeotian to Pontian clayey sediments (ca 6.8–5.2 Ma) (Deshayes 1838; Rousseau 1842; Wenz 1942, 1959; Marinescu 1969; Lubenescu 2016; Popov et al. 2022).

###### Remarks.

Reuss (1868) described *Undulotheca nobilis* based on four specimens (syntypes are available in the GS), but two of them are *Velutinopsis velutina*, since they do not have undulated ribs but smooth shell surface. *Radix kobelti* is not a synonym of *V. velutina*, but it is frequently confused with *V. velutina*. *Radix kobelti* is a shallow-water species of young Pannonian (“Pontian”) sands (e.g. Gorjanović-Kramberger 1923; Neubauer 2023), whereas *V. velutina* is a typical member of Pannonian marls. *Radix kobelti* differs from *V. velutina* in some morphological features, such as not rimmed aperture, open umbo, thickened columellar lip, smaller and less inflated body whorl, thicker shell, and smaller size dimensions. *Velutinopsis kobelti* mentioned by Korpás-Hódi (1983) and *R. kobelti* by Katona et al. (2013) are *V. velutina* specimens indicated also by the recovered associated deep-water mollusc fauna. Marinescu (1969) introduced a new genus (*Velutinellus*) based on the reduced spire and the expanded peristome, but these characteristics are specific for all the species belonging to the genus *Velutinopsis*. Therefore, here we treat the described *Velutinellus pilleus* as a junior synonym of *V. velutina*. *Radix paucispira* name mentioned by Marinescu (1973) and Pană (1975) are probably misidentifications, and it is a young Pannonian shallow-water species. *Velutinopsis simplex* described by Gorjanović-Kramberger is probably a junior synonym of *V. velutina*. The type specimen of *V. simplex* is a poorly preserved specimen wearing a few adequate characters; therefore, it does not support the description of a separate species (Fig. 3B, C). *Velutinopsis simplex* specimens are laterally compacted (Fig. 3B, C, H). “*Lymnocardium*” *margaritaceum* mentioned by Botka et al. (2021) is probably a poorly preserved steinkern of *V. velutina*.

##### Velutinopsis rugosa

Taxon classificationAnimaliaLymnaeidaLymnaeidae

(Gorjanović-Kramberger, 1901)

F4AA2663-B0A4-54CC-8F24-DB6C21884764

Fig. 4A–D

? v 1901 Limnaea amplecta Kramb. Gorj. – Gorjanović-Kramberger, p. 136, pl. X, figs 13–14. [syn. nov.?]
v *1901 Limnaea rugosa Kramb. Gorj. – Gorjanović-Kramberger, p. 138, pl. X, fig. 16.
? *1904 Limnaeus amplecta* Kramberger-Gorjanović – Halaváts, p. 112.
*1904 Limnaeus rugosus* Kramberger-Gorjanović – Halaváts, p. 114.
? *1923 Velutinopsis amplecta* (Gorjanović-Kramberger) – Wenz, pp. 1323–1324. (cum syn.)
*1923 Velutinopsis rugosa* (Gorjanović-Kramberger) – Wenz, p. 1325. (cum syn.)
? v 1923 Hiscerus amplectus Kramb. Gorj. – Gorjanović-Kramberger, pp. 113–114, fig. 3a–b.
v 1944 Velutinopsis rugosa Kr.-G. – Moos, pp. 347–348, pl. XXI, fig. 6.
? v 1944 Hiscerus amplectus Kr.-G. – Moos, p. 373, pl. XXV, fig. 24.
? 1956 Velutinopsis sp. – Papp, p. 71.
v 1967 Velutinopsis rugosa Gorjanović-Kramberger – Taktakishvili, text-fig. 7c. [cop. Gorjanović-Kramberger 1901]
1969 Velutinellus rugosus (Gorjanović-Kramberger) g. n. – Marinescu, pp. 317, 323–325.
? 1969 Velutinellus amplectus (Gorjanović-Kramberger) g. n. – Marinescu, pp. 317, 323–325.
1969 Velutinellus catinus g. n., sp. n. – Marinescu, p. 317, text-figs 3, 4B, pl. I, figs 9a–12b. [syn. nov.?]
? *1974 Hiscerus amplectus* (Gorjanović-Kramberger) – Milan et al., p. 74.
1975 Undulotheca sp. – Pană, pp. 226–227, pl. IV, fig. 13.
1992 Velutinopsis cf. rugosa (Gorj.-Kramb.) – Korpás-Hódi, pl. II, fig. 5.
v 2019 Undulotheca nobilis (Reuss, 1868) – Botka et al., fig. 3: h.

###### Type locality.

Beočin/Beocsin/Belcsény, Fruška Gora, N Serbia (Gorjanović-Kramberger 1901).

###### Type material.

Holotype (by monotypy): SARA Pl.2497.

###### Material examined.

(26 specimens) ***Holotype***. Serbia • 1 spec., holotype; Beočin/Beocsin/Belcsény, Fruška Gora, N Serbia; SARA Pl.2497. ***Lectotype***. Croatia • 1 spec., lectotype of *Hiscerus amplectus*; Zagreb–Gračani, Medvednica Mts., N Croatia; CNHM 5183-348/1-2.

###### Other material.

Serbia • 3 specs; Beočin/Beocsin/Belcsény, Fruška Gora, N Serbia; SARA Pl.2496, Pl.2024.117.1. Croatia • 1 spec.; Grubišno Polje-1 borehole, Sava Basin, N Croatia; CNHM 11. • 2 specs; Ludbreg-2 borehole, Drava Basin, N Croatia; CNHM 10, Moos-51. • 2 specs; Našice/Nekcse, Krndija Mts., N Croatia; HNHM INV 2025.212, INV 2025.218. Hungary • 7 specs, MOL drillcore collection; boreholes: Bajcsa-22, Bajcsa-35, Belezna-20, Inke-15, Inke-24, Mezőcsokonya-Ny-2, Vése-4, Zala Basin, W Hungary; SARA Pl.2024.4.1, Pl.2024.9.1, Pl.2024.13.1, Pl.2024.20.1, Pl.2024.22.1, Pl.2024.29.1, Pl.2024.42.1. • 2 specs, MOL drillcore collection; Ács-1, Gönyü-1, Danube Basin, N Hungary; SARA Pl.2024.1.1, Pl.2024.17.1. Romania • 1 spec.; Tătârlaua/Felsőtatárlaka, Transylvanian Basin, Central Romania; SARA Pl.2024.377.1. • 1 spec.; Jidvei/Zsidve, Transylvanian Basin, Central Romania; SARA Pl.2024.363.1. • 1 spec.; Sângeorgiu de Mureș/Marosszentgyörgy, Transylvanian Basin, Central Romania; SARA Pl.2024.628.1. • 2 specs; Gușterița/Szenterzsébet/Hammersdorf, Transylvanian Basin, Central Romania; HNHM INV 2025.236, INV 2025.244. • 1 spec.; Sighișoara/Segesvár/Schäßburg, Transylvanian Basin, Central Romania; HNHM INV 2025.259. • 1 spec.; Viforoasa/Havadtő, Transylvanian Basin, Central Romania; HNHM INV 2025.254.

###### Note.

For collectors and specimen-related information see Suppl. material 1.

###### Description.

Shell thin, smooth, convex, and globose with dense, concentric, and thickened growth lines or furrows. Dextral shell coiling, three curved whorls rapidly increasing in width. A very strongly widened inflated body whorl with an oval and rimmed aperture. Whorls divided by wide and deep, channel-like sutures. Body whorl fully covers previous whorl and protoconch of spire, it is ~ 8× larger than previous whorl. Apertural edge sharp. Umbo closed in adult specimens. Protoconch could be observed only in well-preserved or in laterally compacted specimens, but no such specimen is currently available. Most characteristics of the species are the same as *V. velutina*. Separation of the two species is possible based on the different ornamentation. Co-occurrence of the two species is observed at some localities (e.g. Gușterița, Transylvanian Basin, Central Romania – Botka et al. 2019). Size parameters: L = 8.5–38.5 mm, W = 12–34.5 mm, L/W = 0.7–1.5, number of furrows = 11–14.

###### Distribution and stratigraphic range.

This species was described and mentioned from old Pannonian offshore (sublittoral and profundal) marls all over the Pannonian Basin: Beočin/Beocsin/Belcsény, Fruška Gora, N Serbia (Gorjanović-Kramberger 1901; Koch 1902); Grubišno Polje-1 borehole, Sava Basin and Ludbreg-2 borehole, Drava Basin (Moos 1944), Zagreb–Kostanjek/Podsused and Zagreb–Gračani, Medvednica Mts. (Papp 1956; this study), Našice/Nekcse, Krndija Mts., N Croatia (this study); Tătârlaua/Felsőtatárlaka and Jidvei/Zsidve (Telegdi-Roth 1909; this study), Sângeorgiu de Mureș/Marosszentgyörgy (Jaskó 1950; this study), Bisericani/Székelyszentlélek (Pană 1975), Gușterița/Szenterzsébet/Hammersdorf, Sighișoara/Segesvár/Schäßburg, and Viforoasa/Havadtő, Transylvanian Basin, Central Romania (Botka et al. 2019; this study); Szombathely-II borehole (Korpás-Hódi 1992), Ács-1 and Gönyü-1 boreholes (this study), Danube Basin, N-NW Hungary; Bajcsa-22, -35, Belezna-20, Inke-15, -24, Mezőcsokonya-Ny-2, and Vése-4 boreholes, Zala Basin, W Hungary (this study). There are some reports from outside the Pannonian Basin, namely from the area of the Eastern Paratethys from the upper Maeotian deposits (Crăguești, Dacian Basin, S Romania – Marinescu 1969). It is a rare member of the “*L.*” *praeponticum* – *R. croatica* mollusc fauna and a less common member of the *Velutinopsis* lineage zone. It is also rare, but present in younger assemblages (*Carinatocongeria digitifera* mollusc biozone as well) together with *V. velutina*. It is present in the assemblages from ca 11.5–3.6 Ma. This species is originated from Lake Pannon, but it successfully migrated into the Eastern Paratethys region in the late Maeotian. It is reported from the *Paradacna*- and *Valenciennius*-bearing deep-water upper Maeotian clayey sediments (ca 6.8–6.1 Ma) (Marinescu 1969; Popov et al. 2022).

###### Remarks.

*Hiscerus amplectus* described by Gorjanović-Kramberger is probably a junior synonym of *Velutinopsis rugosa*. Type specimens of *H. amplectus* are poorly preserved, wearing a few adequate characters; therefore, these do not support description of a separate species (Fig. 4E, F). *Hiscerus amplectus* specimens are less compacted and the protoconch and the first whorls are usually inside the sediment or eroded, therefore cannot be investigated. The lectotype specimen of *H. amplectus* has some thickened growth lines; it may represent a transitional form between *V. velutina* and *V. rugosa*. Specimens described and illustrated by Papp (1956), Pană (1975), and Botka et al. (2019) most probably belong to *V. rugosa* based on their thickened growth lines or furrows, which are less developed unlike in the case of *Undulotheca*. This is a transitional form between *V. velutina* and *U. nobilis*. Marinescu (1969) introduced a new genus (*Velutinellus*) for the Dacian Basin lymnaeids and assigned *V. rugosa* into this genus based on the reduced spire and the expanded peristome, but these characteristics are specific for all the species belonging to the genus *Velutinopsis*. *Velutinellus* is not accepted here because of the lack of any significant distinctive morphological character, and *Velutinellus catinus* is also treated as a junior synonym of *V. rugosa*.

##### Genus Undulotheca

Taxon classificationAnimaliaLymnaeidaLymnaeidae

Gorjanović-Kramberger, 1923

4DAB69F5-4A52-5959-8842-35885619200C

###### Type species.

*Undulotheca nobilis* (Reuss, 1868); subsequent designation.

###### Original description.

“This genus possesses thin, large, smooth, or somewhat inflated, rounded to oval shells with strong, concentric, undulated ribs, apart from the posterior edge. Body whorl is always widened and covers the spire. The spire consists of a few whorls reaching beyond the posterior edge of the shell, where the peristome – beside the spire – partially turns back and can create a columellar lip. The shell is dextral or sinistral coiled. The shell is evolute leaving behind a suture edge, which can be observed at the provalencienniids and valencienniids as well. The undulated ribs are starting to bend in on the right side of the shell, which is the first proof of the future siphonal canal observed at the provalencienniids” (in German in Gorjanović-Kramberger 1923, translated by D. Botka).

###### Emended diagnosis.

Shell thin, smooth, convex, and globose with dense, concentric, and undulated ribs. Furrows and growth lines can be observed between ribs. Specimens preserved with their original shell show a reddish-brown colour. Dextral shell coiling, two curved whorls rapidly increasing in width and a very strongly widened inflated body whorl with an oval and rimmed aperture. Whorls divided by wide and deep, channel-like sutures. Body whorl fully covers previous whorl and protoconch of spire (convolute shell). Apertural edge sharp. Umbo closed in adult specimens. Protoconch could be observed only in well-preserved laterally compacted specimens, it is simple and smooth.

##### Undulotheca nobilis

Taxon classificationAnimaliaLymnaeidaLymnaeidae

(Reuss, 1868)

FDEA229E-5371-552A-98A3-56F3A01B7B2E

Fig. 5A–J

v *1868 Limnaeus nobilis n. sp. – Reuss, pp. 85–87, pl. II, figs 1a–2b.
1899 (?) Limnaeus nobilis Reuss – Gorjanović-Kramberger, p. 127, pl. V, fig. 2.
? v 1901 Limnaea undulata Kramb. Gorj. – Gorjanović-Kramberger, p. 136, pl. X, fig. 12. [syn. nov.?]
v *1904 Limnaeus nobilis* Reuss – Halaváts, p. 114.
? *1904 Limnaeus undulatus* Kramberger-Gorjanović – Halaváts, p. 115.
*1923 Velutinopsis nobilis* (Reuss) – Wenz, p. 1324. (cum syn.)
? *1923 Velutinopsis undulata* (Gorjanović-Kramberger) – Wenz, p. 1325. (cum syn.)
? v 1923 Hiscerus undulatus Kramb. Gorj. – Gorjanović-Kramberger, p. 113, fig. 4.
v 1944 Velutinopsis conf. *nobilis* Reuss – Moos, pp. 352–353, pl. XXI, fig. 7.
v 1944 Velutinopsis transiens n. sp. – Moos, pp. 353–355, pl. XXII, figs 10–10a. [syn. nov.]
? v 1944 Hiscerus (Velutinopsis) undulatus Kr.-G. – Moos, pp. 373–374, pl. XXV, fig. 25.
v 1967 Velutinopsis nobilis (Reuss) – Taktakishvili, text-fig. 8a–b. [cop. Reuss 1868]
1969 Velutinellus transiens (Moos) g. n. – Marinescu, pp. 317, 323–325.
? v *1974 Hiscerus undulatus* (Gorjanović-Kramberger) – Milan et al., p. 74.
v *1974 Velutinopsis transiens* Moos – Milan et al., p. 146.
1975 Undulotheca sp. – Pană, pp. 226–227, pl. IV, fig. 12.
v 1976 Neodelminiella venusta n. gen., n. sp. – Kochansky-Devidé and Pikija, pp. 401, 405–406, pl. I, fig. 4a–b. [syn. nov.]
1977 Undulotheca pancici pancici (Brusina) – Lubenescu and Lubenescu, pl. II, figs 12–13.
v 1981 Neodelminiella venusta Kochansky-Devide and Pikija – Sremac, pp. 115–116, 121, pl. I, figs 23–25.
1981 Undulotheca pancici (Brusina, 1892) – Lubenescu, p. 182, pl. XIV, figs 1–3.
2011 Undulotheca pancici – Rundić et al., fig. 8B.
non v 2019 Undulotheca nobilis (Reuss, 1868) – Botka et al., fig. 3h. [= Velutinopsis rugosa Gorjanović-Kramberger]
v 2019 Undulotheca nobilis (Reuss, 1868) – Botka et al., fig. 3: i.

###### Type locality.

Şura Mare/Nagycsűr–Hamba/Kakasfalva/Hahnbach, Transylvanian Basin, Central Romania (Reuss 1868). Originally, four specimens were collected by Reuss (1868) from the quarry of Hamba/Kakasfalva/Hahnbach, between Sibiu/Nagyszeben/Hermannstadt and Mediaș/Medgyes/Mediasch, south of Agârbiciu/Szászegerbegy/Arbegen.

###### Type material.

Lectotype: 1 specimen, GS 1868/005/0001/1. Originally, Reuss (1868) described *Limnaeus nobilis* based on four specimens, but two steinkerns represent *Velutinopsis velutina* (paralectotypes, GS 1868/005/0001), since they do not have undulated ribs but smooth shell surface. He collected and figured two *Undulotheca nobilis* specimens as well, but one of them is missing from the collection. The remaining specimen was designated as a lectotype.

###### Material examined.

(88 specimens) ***Lectotype***. Romania • 1 spec., lectotype; Şura Mare/Nagycsűr–Hamba/Kakasfalva/Hahnbach, Transylvanian Basin, Central Romania; GS 1868/005/0001/1. ***Holotype***. Croatia • 1 spec., holotype+genotype (by monotypy) of *Hiscerus undulatus*; Novi Marof, Zagorje Basin, N Croatia; CNHM 5348-501. • 1 spec., holotype+genotype of *Neodelminiella venusta*; Zagreb–Vugrovec, Medvednica Mts., N Croatia; CNHM 983. • 1 spec., holotype (by monotypy) of *Velutinopsis transiens*; Grubišno Polje-1 borehole, Sava Basin, N Croatia; CNHM 12.

###### Other material.

Romania • 11 specs; Slimnic/Szelindek, Transylvanian Basin, Central Romania; SARA Pl.1800, Pl.1881. • 8 specs; Axente Sever/Asszonyfalva, Transylvanian Basin, Central Romania; SARA Pl.1811, Pl.2024.203.1, Pl.2024.204.1, Pl.2024.214.1, Pl.2024.215.1, Pl.2024.216.1, Pl.2024.226.1, Pl.2024.230.1. • 2 specs; Meșcreac/Meggykerék, Transylvanian Basin, Central Romania; SARA Pl.2024.468.1, Pl.2024.469.1. • 1 spec.; Șona/Szépmező, Transylvanian Basin, Central Romania; SARA Pl.2024.365.1. • 1 spec.; Galda de Jos/Alsógáld, Transylvanian Basin, Central Romania; SARA Pl.2024.591.1. • 1 spec.; Corund/Korond, Transylvanian Basin, Central Romania; BBU 2123. • 2 specs; Gușterița/Szenterzsébet/Hammersdorf, Transylvanian Basin, Central Romania; BM49.403, 49.431. • 16 specs; Mihalț/Mihálcfalva, Transylvanian Basin, Central Romania; HNHM INV 2025.233. • 25 specs; Tău/Székástóhát, Transylvanian Basin, Central Romania; HNHM INV 2025.231. • 1 spec.; Daia Română/Oláhdálya, Transylvanian Basin, Central Romania; HNHM INV 2025.260. • 1 spec.; Agârbiciu/Szászegerbegy, Transylvanian Basin, Central Romania; HNHM INV 2025.235. • 1 spec.; Oarba de Mureș/Marosorbó, Transylvanian Basin, Central Romania; HNHM INV 2025.228. Serbia • 1 spec.; Beočin/Beocsin/Belcsény, Fruška Gora, N Serbia; SARA Pl.2498. Croatia • 1 spec.; Zagreb–Vugrovec, Medvednica Mts., N Croatia; CNHM 1159. • 2 specs; Zagreb–Čučerje, Medvednica Mts., N Croatia; CNHM 984a. • 2 specs; Brodski Zdenci, Dilj Mts., N Croatia; CNHM 1162. • 2 specs; Gojlo-25 and Osekovo-1 boreholes, Sava Basin, N Croatia; CNHM 13, Moos-52. • 1 juvenile spec.; Gojlo-4 borehole, Sava Basin, N Croatia; CNHM 14. • 4 specs; Našice/Nekcse, Krndija Mts., N Croatia; HNHM INV 2025.213, INV 2025.219. Hungary • 1 spec., MOL drillcore collection; Zákány-2 borehole, Drava Basin, W Hungary; SARA Pl.2024.45.1.

###### Note.

For collectors and specimen-related information see Suppl. material 1.

###### Description.

Shell thin, smooth, convex, and globose with dense, concentric, and undulated ribs. Furrows and growth lines can be observed between ribs. Specimens preserved with their original shell show a reddish-brown colour. Dextral shell coiling, two curved whorls rapidly increasing in width and a very strongly widened inflated body whorl with an oval and rimmed aperture. Whorls divided by wide and deep, channel-like sutures. Body whorl fully covers previous whorl and protoconch of spire, it is ~ 8× larger than previous whorl. Apertural edge sharp. Umbo closed in adult specimens. Protoconch could be observed only in well-preserved laterally compacted specimens, it is simple and smooth. This species is sometimes confused with *U. pancici*. One might think that *U. nobilis* specimens are juvenile specimens of *U. pancici*, but latter species bears wider ribs, and its larger shell has a more rounded outline (see L/W ratios). They do not always occur at the same localities or layers as well. Size parameters: L = 20.5–42 mm, W = 14.5–34 mm, L/W = 0.95–1.8, NR = 17–23, WR = 0.5–1.5 mm, WSR = 0.5–1.0 mm.

###### Distribution and stratigraphic range.

This species was described and mentioned from old Pannonian offshore (sublittoral and profundal) marls all over the Pannonian Basin: Şura Mare/Nagycsűr–Hamba/Kakasfalva/Hahnbach (Reuss 1868); Slimnic/Szelindek (Halaváts 1912); Meșcreac/Meggykerék, Galda de Jos/Alsógáld, Șona/Szépmező, and Axente Sever/Asszonyfalva (Telegdi-Roth 1903, 1907, 1909; this study); Bisericani/Székelyszentlélek (Pană 1975); Valea Rodului and Amnaș-Aciliu (Lubenescu and Lubenescu 1977; Lubenescu 1981); Gușterița/Szenterzsébet/Hammersdorf (Lubenescu and Lubenescu 1977; Lubenescu 1981; Botka et al. 2019); Corund/Korond, Mihalț/Mihálcfalva, Tău/Székástóhát, Daia Română/Oláhdálya, Agârbiciu/Szászegerbegy, and Oarba de Mureș/Marosorbó (this study), Transylvanian Basin, Central Romania; Beočin/Beocsin/Belcsény, Fruška Gora, N Serbia (Koch 1902); PdUS-7 borehole in Belgrade, Šumadija Hills, Central Serbia (Rundić et al. 2011); Novi Marof, Zagorje Basin (Gorjanović-Kramberger 1901); Londžica/Babindol, Krndija Mts. (Gorjanović-Kramberger 1899); Grubišno Polje-1, Osekovo-1, Gojlo-4, and Gojlo-25 boreholes, Sava Basin (Moos 1944); Zagreb–Vugrovec and Zagreb–Čučerje, Medvednica Mts. (Kochansky-Devidé and Pikija 1976; Sremac 1981); Brodski Zdenci, Dilj Mts. (Sremac 1981); Našice/Nekcse, Krndija Mts., N Croatia (this study); Zákány-2 borehole, Drava Basin, W Hungary (this study). It is a common member of the *Congeria banatica* mollusc fauna. Based on the FAD of *U. nobilis* and co-occurrence with *V. velutina*, *V. rugosa*, and *U. pancici*, an *Undulotheca* lineage zone is proposed (ca 11.2–10.5 Ma). LAD of this species is ca 10.5 Ma.

###### Remarks.

*Lymnaea undulata* described by Gorjanović-Kramberger (1901) is probably a junior synonym of *Undulotheca nobilis* based on the characteristics of the holotype material. It is a poorly preserved specimen, with a missing protoconch (Fig. 5E). Later, it was assigned into the genus *Hiscerus* (Gorjanović-Kramberger 1923), but still based on this single, broken specimen. Moos (1944) also found a similar specimen, but he mentioned in his description that it could be a juvenile of a more developed valencienniid. *Velutinopsis transiens* described by Moos (1944) is also a junior synonym of *U. nobilis* based on the studied holotype material. It is a poorly preserved and poorly prepared specimen (Fig. 5D). Moos (1944) classified it as a new transitional species between *Velutinopsis* and *Undulotheca* based on its undulated ribs. Marinescu (1969) placed this species into the genus *Velutinellus* based on the reduced spire and the expanded peristome. These characteristics, however, are typical for all the species belonging to the genera *Velutinopsis* and *Undulotheca*. *Neodelminiella venusta* described by Kochansky-Devidé and Pikija (1976) can be identified with *U. nobilis* based on their shell’s characteristics (Fig. 5H). They introduced a new genus (*Neodelminiella*) based on the similarities to the Middle Miocene *Delminiella*. Lubenescu and Lubenescu (1977) and Lubenescu (1981) described the old Pannonian mollusc fauna of the Transylvanian Basin and consistently used the name *U. pancici* for *U. nobilis*, however, *U. pancici* is a more developed form and only present at some localities in the Transylvanian Basin. A specimen described and figured from the middle part of the Gușterița clay pit sequence by Botka et al. (2019: fig. 3h) represents a poorly preserved *V. rugosa*.

##### Undulotheca pancici

Taxon classificationAnimaliaLymnaeidaLymnaeidae

(Brusina, 1893)

C4F42262-7803-54C8-97EC-B1ADB75F368C

Fig. 6A–L

v *1893 *Limnaea Pančići* Brusina – Brusina, pp. 43–44.
v 1901 *Limnaea Pančići* Brusina – Gorjanović-Kramberger, pp. 136–137, pl. X, figs 9–10.
v 1901 *Limnaea Halavátsi* Kramb. Gorj. – Gorjanović-Kramberger, pp. 137–138, pl. X, fig. 11. [syn. nov.]
v 1902 *Velutinopsis Pančići* Brus. – Brusina, pl. I, figs 28–29.
*1904 Limnaeus Halavátsi* Kramberger-Gorjanović – Halaváts, p. 113.
*1904 Limnaeus Panćići* [sic!] Kramberger-Gorjanović – Halaváts, p. 114.
*1923 Velutinopsis halavátsi* (Gorjanović-Kramberger) – Wenz, p. 1324. (cum syn.)
*1923 Velutinopsis pančiči* [sic!] Brusina – Wenz, p. 1325. (cum syn.)
v 1923 *Undulotheca Pančići* Brus. sp. – Gorjanović-Kramberger, p. 97, text-fig. 4a.
v 1923 *Undulotheca Halavátsi* Kramb. Gorj. – Gorjanović-Kramberger, pp. 97–98, text-fig. 4b.
v 1923 Undulotheca rotundata Kramb. Gorj. – Gorjanović-Kramberger, p. 98, text-fig. 4d. [syn. nov.]
v 1923 *Undulotheca Kochi* Kramb. Gorj. – Gorjanović-Kramberger, p. 98, text-fig. 4c, fig. 1. [syn. nov.]
v 1944 Undulotheca gojlo n. sp. – Moos, pp. 348–350, pl. XXI, figs 8–8a. [syn. nov.]
v 1944 Undulotheca rotundata Kr.-G. – Moos, pp. 350–352, pl. XXI, figs 9–9a.
v 1959 Valenciennius (Undulotheca) pančići (Brusina) – Wenz, p. 96, text-fig. 308. [cop. Brusina 1902]
v 1967 *Undulotheca pančiči* [sic!] (Brusina) – Taktakishvili, p. 15, text-fig. 10a, pl. I, figs 1–2. [cop. Brusina 1902]
v 1967 Undulotheca halavátsi Gorjanović-Kramberger – Taktakishvili, p. 16, text-fig. 10b, pl. I, fig. 3. [cop. Gorjanović-Kramberger 1901]
v 1967 Undulotheca kochi Gorjanović-Kramberger – Taktakishvili, p. 16, text-fig. 10c, pl. I, fig. 4. [cop. Gorjanović-Kramberger 1923]
v 1967 Undulotheca rotundata Gorjanović-Kramberger – Taktakishvili, p. 17, text-fig. 10d, pl. I, figs 5–6. [cop. Gorjanović-Kramberger 1901]
v 1967 *Udulotheca* [sic!] gojlo Moos – Taktakishvili, pp. 17–19, pl. II, fig. 1. [cop. Moos 1944]
v *1974 Undulotheca gojlo* Moos – Milan et al., p. 139.
v *1974 Undulotheca kochi* Gorjanović-Kramberger – Milan et al., p. 139.
v *1974 Udulotheca* [sic!] pancici (Brusina) – Milan et al., p. 146.
1975 Undulotheca sp. – Pană, pp. 226–227, pl. IV, fig. 14.
v 1976 Neodelminiella lucinoides n. gen., n. sp. – Kochansky-Devidé and Pikija, pp. 402, 406, pl. I, fig. 3a–b. [syn. nov.]
non 1977 Undulotheca pancici pancici (Brusina) – Lubenescu and Lubenescu, pl. II, figs 12–13. [= Undulotheca nobilis (Reuss)]
1978 Undulotheca pančići (Brus.) – Stevanović, pp. 328–330, pl. VI, figs 1–4.
1981 Undulotheca rotundata (Gorjanović-Kramberger, 1901) – Lubenescu, p. 182.
non 1981 Undulotheca pancici (Brusina, 1892) – Lubenescu, p. 182, pl. XIV, figs 1–3. [= Undulotheca nobilis (Reuss)]
? v 1981 Undulotheca cf. gojlo Moos – Sremac, pp. 114–115, 120, pl. I, fig. 20.
v 1981 Neodelminiella lucinoides Kochansky-Devide and Pikija – Sremac, pp. 115, 120–121, pl. I, figs 21–22.
1985 Undulotheca halavatsi Koch [sic!] – Stevanović and Papp, pl. 27, fig. 12.
non 2011 Undulotheca pancici – Rundić et al., fig. 8: B. [= Undulotheca nobilis (Reuss)]
2013 Undulotheca halavaci [sic!] Koch [sic!] – ter Borgh et al., fig. 9: 15.
2013 Provalenciennesia sp. – ter Borgh et al., fig. 9: 16.
v 2019 Undulotheca halavatsi Gorjanović-Kramberger, 1901 – Botka et al., fig. 3: j.
2021 Undulotheca halavatsi Gorjanović-Kramberger, 1901 – Chira et al., fig. 5: j–k.
v 2023 Undulotheca halavatsi (Gorjanović-Kramberger, 1901) – Neubauer, fig. 6.7: a [cop. Botka et al. 2019]

###### Type locality.

Jantalova pećina, Zagreb–Markuševec, Medvednica Mts., N Croatia (Brusina 1893).

###### Type material.

Holotype (by monotypy): CNHM 5771-921.

###### Material examined.

(62 specimens) ***Holotype***. Croatia • 1 spec., holotype of *Undulotheca pancici*; Zagreb–Markuševec, Medvednica Mts., N Croatia; CNHM 5771-921. • 1 spec., holotype (by monotypy) of *Undulotheca kochi*; Bektež, Krndija Mts., N Croatia; CNHM 5776-926. • 2 specs, holotype (by monotypy) of *Undulotheca gojlo*; Gojlo-4 borehole, Sava Basin, N Croatia; CNHM 18/1-2. • 1 spec., holotype of *Neodelminiella lucinoides*; Voćarica, Sava Basin, N Croatia; CNHM 984. Romania • 1 spec., holotype (by monotypy) of *Undulotheca halavatsi*; Vârciorova/Varcsaró, Godjan Mts., W Romania; SARA Pl.128.

###### Other material.

Romania • 6 specs; Vârciorova/Varcsaró, Godjan Mts., W Romania; SARA Pl.91, Pl.1869, Pl.6370, Pl.2024.123.1, Pl.2024.124.1, Pl.5760. • 2 specs; Chelința/Kelence, Şimleu Basin, NW Romania; SARA Pl.2211. • 1 spec.; Șeica Mare–Calvasăr/Nagyselyk–Hidegvíz, Transylvanian Basin, Central Romania; SARA Pl.2024.177.1. • 11 specs; Gușterița/Szenterzsébet/Hammersdorf, Transylvanian Basin, Central Romania; BBU 7645, 7653/1, 7653/2; BM49.373, 49.382, 49.383, 49.384, 49.445, 49.448, 49.449, 49.450, 49.638. • 16 specs; Gușterița/Szenterzsébet/Hammersdorf, Transylvanian Basin, Central Romania; HNHM INV 2025.238 to INV 2025.243, INV 2025.251. Croatia • 1 spec.; Zagreb–Vugrovec, Medvednica Mts., N Croatia; CNHM 1167. • 4 specs; Zagreb–Borčec, Medvednica Mts., N Croatia; CNHM 5772-922/1-4. • 1 spec.; Vojnovec Kalnički, Kalnik Mts., N Croatia; CNHM 5775-925. • 1 spec.; Hrkanovci Đakovački, N Croatia; CNHM 5773-923. • 1 spec.; Slavonski Brod–Duboki potok, Dilj Mts., N Croatia; CNHM 16. • 4 specs; Našice/Nekcse, Krndija Mts., N Croatia; HNHM INV 2025.221, INV 2025.222, INV 2025.224. Serbia • 6 specs; Beočin/Beocsin/Belcsény, Fruška Gora, N Serbia; CNHM 15, 17/1-3; SARA Pl.2024.118.1; NHMB 1762. HUNGARY • 2 specs, MOL drillcore collection; Bajánsenye-1 borehole, Zala Basin, W Hungary; SARA Pl.2024.2.1. • 1 spec., MOL drillcore collection; Tura-3 borehole, Zagyva Basin, N Hungary; SARA Pl.2024.61.1.

###### Note.

For collectors and specimen-related information see Suppl. material 1.

###### Description.

Shell thin, smooth, convex, and globose with dense, concentric, undulated, and strong ribs. These ribs become narrower towards edges of shell. On posterior edge of shell, ribs extend at a steep to vertical angle, while on anterior part of shell, they bend back in a curved shape towards protoconch. Furrows and growth lines can be observed between ribs. Specimens preserved with their original shell show a reddish-brown colour. Dextral shell coiling, two curved whorls rapidly increasing in width and a very strongly widened inflated body whorl with an oval and rimmed aperture. Apertural rim wide and flat and can be followed around entire aperture. Whorls divided by wide and deep, channel-like sutures. Body whorl fully covers previous whorl and protoconch of spire, it is ~ 8–10× larger than previous whorl. Umbo closed in adult specimens. Protoconch could be observed only in well-preserved laterally compacted specimens, it is simple and smooth. This species is sometimes confused with *U. nobilis*. Latter species bears narrower ribs, and its shell has a less rounded and more elongated outline (see L/W ratios). Juvenile specimens are difficult to classify as *U. nobilis* or *U. pancici*, since they do not wear adequate characters, they do not have developed shell outline nor ribs. Size parameters: L = 25.5–70 mm, W = 24.5–73 mm, L/W = 0.8–1.25, NR = 16–29, WR = 1.0–2.6 mm, WSR = 1.0–2.5 mm.

###### Distribution and stratigraphic range.

This species was described and mentioned from old Pannonian offshore (sublittoral and profundal) marls all over the Pannonian Basin: Gârbova/Szászorbó, Valea Rodului, Măgură Copacului, Sibiu–Turnișor/Nagyszeben–Kistorony (Lubenescu 1981); Șeica Mare–Calvasăr/Nagyselyk–Hidegvíz (Telegdi-Roth 1910; this study); Bisericani/Székelyszentlélek (Pană 1975); Gușterița/Szenterzsébet/Hammersdorf (Lubenescu 1981; Botka et al. 2019; Chira et al. 2021; this study), Transylvanian Basin, Central Romania; Belgrade–Lomina (Brusina 1893); Belgrade–Terazije, Šumadija Hills, Central Serbia (Stevanović 1978); Beočin/Beocsin/Belcsény, Fruška Gora, N Serbia (Koch 1902; Moos 1944; Stevanović and Papp 1985; ter Borgh et al. 2013; this study); Zagreb–Borčec (Gorjanović-Kramberger 1901, 1923); Zagreb–Markuševec (Brusina 1893, 1902); Zagreb–Vugrovec (Sremac 1981), Medvednica Mts.; Gojlo-4 borehole (Moos 1944) and Voćarica, Sava Basin (Kochansky-Devidé and Pikija 1976); Vojnovec Kalnički, Kalnik Mts. (Gorjanović-Kramberger 1923); Busnovi, Požeška Gora (Sremac 1981); Slavonski Brod­–Duboki potok (Moos 1944) and Hrkanovci Đakovački, Dilj Mts. (Gorjanović-Kramberger 1923); Bektež (Gorjanović-Kramberger 1923) and Našice/Nekcse, Krndija Mts., N Croatia (this study); Vârciorova/Varcsaró, Godjan Mts., W Romania (Gorjanović-Kramberger 1901, 1923); Chelința/Kelence, Şimleu Basin, NW Romania (this study); Bajánsenye-1 borehole, Zala Basin, W Hungary (this study); Tura-3 borehole, Zagyva Basin, N Hungary (this study). It is a common member of the *Undulotheca* lineage zone (ca 11.2–10.5 Ma). FAD of this species is ca 10.7 Ma, while its LAD is ca 10.5 Ma.

###### Remarks.

*Undulotheca halavatsi* described by Gorjanović-Kramberger (1901) is a sinistrally (Fig. 6G) coiled and poorly preserved specimen showing the inner side of the shell, which is obviously identical with *Undulotheca pancici*, since it bears the same characteristics. Gorjanović-Kramberger (1923) defined *Undulotheca rotundata* as a transitional form between *U. pancici* and *Provalenciennesia limnaeoidea* based on the location of its spire and shell outline. He noted that it was interesting to find different evolutionary stages of forms in the same stratigraphic horizon. A slightly laterally compacted specimen was described as *Undulotheca kochi* by Gorjanović-Kramberger (1923) based on its elongated outline, number of ribs, and the location of its spire. Moos (1944) described *Undulotheca gojlo* from a drillcore (Gojlo-4 borehole) based on its elongated outline and low number (12) of ribs compared to other *Undulotheca* species (Fig. 6D, E). He hypothesised that it is the most primitive form of *Undulotheca* genus, however, such an interpretation is hardly supported by a single, poorly preserved (laterally compressed and incomplete) specimen. Kochansky-Devidé and Pikija (1976) created a new genus for cap-like snails of Lake Pannon, *Neodelminiella*, because of its relation to the Middle Miocene *Delminiella* genus known from the Dinaride Lake System (Kochansky-Devidé and Slišković 1972; Harzhauser et al. 2016). Their Pannonian species, however, are identical with the earlier described *Undulotheca* species (Fig. 6F). Sremac (1981) separated a poorly preserved specimen as *U. cf. gojlo*. Lubenescu and Lubenescu (1977) and Lubenescu (1981) recognised two *Undulotheca* species in the Transylvanian Basin; they used *U. pancici* for the smaller and *U. rotundata* for the larger *Undulotheca* species. *Provalenciennesia* sp. depicted by ter Borgh et al. (2013: fig. 9: 16) is an *Undulotheca* specimen since its spire is located on the rim of the shell.

#### Valencienniinae Gorjanović-Kramberger, 1923 syn. nov. (= Amphipepleinae Pini, 1877)

**Notes**. In the following section, we discuss the original description of Valencienniinae and the taxonomic issues associated with this clade. Valencienniins possess unusual morphological characters and may also have had distinctive palaeoecological requirements among Lymnaeidae. However, because of the presence of transitional forms, we do not consider it justified to treat them as a separate subfamily. Therefore, we regard Valencienniinae as a synonym of Amphipepleinae. In this paper and in Suppl. material 1, we use the terms ‘valencienniins’ or *Provalenciennesia*/*Valenciennius* sp. to refer to this clade.

**Original description**. “The reasons that led me to split the *Valenciennesia* into two genera are of a stratigraphic and genetic nature. This latter aspect is particularly evident in the older group – the *Provalenciennesia* – because it represents a true transitional group in which it is difficult to separate certain forms from those that precede them – the *Undulotheca* species – or follow them – the *Valenciennesia* species. It should also be noted that not a single representative of the older, i.e. the Lower Pontian valenciennesiids reach up into the Upper Pontian formations and that this older Pontian part can therefore be described as the ‘*Provalenciennesia* Horizon’ and the younger one as the ‘*Valenciennesia* Horizon’” (in German in Gorjanović-Kramberger 1923, translated by D. Botka). [It was originally described as a subfamily under the name of Valenciennesiidae].

**Remarks**. The original diagnosis of Gorjanović-Kramberger (1923) does not contain much information on the morphology of the introduced subfamily. Valencienniins are characterised by large size (≤10–15 cm in length and width), reduced coiling (1–2 whorls), wide oval or elongated outline and aperture, coiling present not on marginal part of shell, ornamentation with 2–3 mm wide, concentric, undulated, rounded or sharp ribs, siphonal canal or an undulated pre-siphonal structure present. These characters are atypical for Lymnaeidae and are difficult to delimit. Given the occurrence of transitional forms, it is highly doubtful that an artificial boundary can be justified within this lineage to support Valencienniinae as a distinct clade.

##### Genus Provalenciennesia

Taxon classificationAnimaliaLymnaeidaLymnaeidae

Gorjanović-Kramberger, 1923

B37972DC-B98D-5ECA-88D2-5788ECB87C28

###### Synonym.

*Provalenciennius* [sic!].

###### Type species.

*Provalenciennesia pauli* (R. Hörnes, 1875); subsequent designation.

###### Original description.

“As already mentioned, the representatives of this subgenus developed from *Undulotheca* by the fact that the coiling gradually rose above the posterior margin of the shell gradually lifted and shifted forwards, whereby the posterior margin became free and elongated; at the same time the undulated ribs began to bend in slightly on the right side of the coiling. In front of this siphonal sinus, some rib bends are still visible on the posterior margin, which are to be regarded as the remains of the sutural edge of the last whorl” (in German in Gorjanović-Kramberger 1923, translated by D. Botka). [It was originally described as a subgenus].

###### Emended diagnosis.

Members of the genus *Provalenciennesia* are characterised by large size (≤10–15 cm in length and width), reduced coiling (2 whorls), wide oval or elongated outline and aperture, coiling present not on marginal part of shell, ornamentation with 2–3 mm wide, concentric, undulated, rounded or sharp ribs, an undulated pre-siphonal structure present instead of a siphonal canal.

##### Provalenciennesia limnaeoidea

Taxon classificationAnimaliaLymnaeidaLymnaeidae

(Gorjanović-Kramberger, 1901)

C11B598A-7A52-5F96-8EF7-DF5DFF8E83D1

Fig. 7A–G

v *1901 Valenciennesia limnaeoidea Kramb. Gorj. – Gorjanović-Kramberger, p. 135, text-fig. 3, pl. X, figs 5–7.
*1904 Valenciennesia limnaeoidea* Kramberger-Gorjanović – Halaváts, p. 124.
*1923 Valenciennesia limnaeoidea* Gorjanović-Kramberger – Wenz, p. 1332. (cum syn.)
v 1923 Provalenciennesia limnaeoidea K. G. – Gorjanović-Kramberger, p. 99, text-fig. 1b.
v 1923 *Provalenciennesia Poljaki* Kramb. Gorj. – Gorjanović-Kramberger, pp. 100–101, fig. 2. [syn. nov.]
v 1944 Provalenciennesia limnaeoidea Kr.-G. – Moos, p. 355, pl. XXII, fig. 11.
v 1967 Provalenciennesia poljaki Gorjanović-Kramberger – Taktakishvili, pp. 21–22, pl. II, fig. 5. [cop. Gorjanović-Kramberger 1923]
v 1967 Provalenciennesia limnaeoidea Gorjanović-Kramberger – Taktakishvili, pp. 24–25, pl. III, figs 4–6. [cop. Gorjanović-Kramberger 1901]
v *1974 Provalenciennesia limnaeoidea* (Gorjanović-Kramberger) – Milan et al., p. 130.
v *1974 Provalenciennesia poljaki* Gorjanović-Kramberger – Milan et al., p. 131.

###### Type locality.

Zagreb–Bačun (Borje), Medvednica Mts., N Croatia (Gorjanović-Kramberger 1901).

###### Type material.

Lectotype: CNHM 5768-918 and paralectotype: CNHM 5767-917 (new designations). Milan et al. (1974) incorrectly designated the specimen CNHM 5767-917 as holotype of *Provalenciennesia limnaeoidea*; therefore, specimens CNHM 5767-917 and 5768-918 remained syntypes. Specimen CNHM 5768-918 has a well-preserved protoconch, therefore, here we designate it as lectotype.

###### Material examined.

(5 specimens) ***Lectotype***. Croatia • 1 spec., lectotype; Zagreb–Bačun (Borje), Medvednica Mts., N Croatia; CNHM 5768-918. ***Paralectotype***. Croatia • 1 spec., paralectotype; Zagreb–Bačun (Borje), Medvednica Mts., N Croatia; CNHM 5767-917. ***Holotype***. Croatia • 1 spec., holotype (by monotypy) of *Provalenciennesia poljaki*; Zagreb–Bačun (Borje), Medvednica Mts., N Croatia; CNHM 5770-920.

###### Other material.

Croatia • 1 spec.; Daruvar/Daruvár, Papuk Mts., N Croatia; CNHM 5766-916. Serbia • 1 spec.; Beočin/Beocsin/Belcsény, Fruška Gora, N Serbia; ELTE G-40.

###### Note.

For collectors and specimen-related information see Suppl. material 1.

###### Description.

Shell thin, smooth, convex, and flat with dense, concentric, undulated, and strong ribs. These ribs become narrower towards edges of shell. On posterior edge of shell, ribs extend at a steep to vertical angle, while on anterior part of shell, they bend back in a curved shape towards protoconch. Furrows and growth lines can be observed between ribs. Dextral shell coiling, two curved whorls rapidly increasing in width and a very strongly widened inflated body whorl with an oval and rimmed aperture. Apertural rim wide and flat and can be followed around the entire aperture. Whorls divided by wide and deep, channel-like sutures. Body whorl fully covers previous whorl and protoconch of spire, it is ~ 8–10× larger than previous whorl. Spire displaced from posterior edge towards centre of shell. Umbo closed in adult specimens. A slight folding of ribs can be observed on dextral side of shell, which is precursor of siphonal canal. Protoconch could be observed only in well-preserved laterally compacted specimens, it is simple and smooth. It is a transitional form between *Undulotheca pancici* and other *Provalenciennesia* species. Size parameters: L = 66–72.5 mm, W = 50–73 mm, L/W = 0.95–1.35, NR = 24–31, WR = 1.0–1.5 mm, WSR = 1.5–2.0 mm.

###### Distribution and stratigraphic range.

This species was described and mentioned from a few localities of old Pannonian offshore (sublittoral and profundal) marls of the Pannonian Basin: Zagreb–Bačun (Borje), Medvednica Mts. (Gorjanović-Kramberger 1901, 1923); Daruvar/Daruvár, Papuk Mts. (this study); Bednja-5 borehole, Sava Basin, N Croatia (Moos 1944); Beočin/Beocsin/Belcsény, Fruška Gora, N Serbia (this study). It is a rare member of the *Congeria banatica* and the *Carinatocongeria digitifera* deep-water mollusc fauna. Uppermost part of the *Congeria banatica* and lowermost part of the *Carinatocongeria digitifera* mollusc biozone. FAD of this species is ca 10.5 Ma, while its LAD is ca 9.8 Ma. Based on the FAD of *P. limnaeoidea* and co-occurrence with *U. pancici* and other provalencienniids, *Provalenciennesia* lineage zone is proposed correlated to the uppermost part of *C. banatica* profundal mollusc biozone (ca 10.5–9.6 Ma).

###### Remarks.

Moos (1944) reported a fragmentary specimen as *Provalenciennesia limnaeoidea* from Bednja-5 borehole, but that specimen does not wear adequate characters to classify it even as a *Provalenciennesia* species. *Provalenciennesia poljaki* described by Gorjanović-Kramberger (1923) is a junior synonym (Fig. 7C–E) of *P. limnaeoidea* since its holotype bears the same characters and it is probably originated from the same locality as the lectotype of *P. limnaeoidea* (Fig. 7A, B). General shape is the only difference between the two holotype specimens, *P. limnaeoidea* being somewhat more elongated than *P. poljaki*.

##### Provalenciennesia arthaberi

Taxon classificationAnimaliaLymnaeidaLymnaeidae

(Gorjanović-Kramberger, 1901)

6032CEE4-16C1-5B1B-B1E0-574FBD2EB402

Fig. 8A–N

1868 Valenciennesia annulata Rousseau – Reuss, pp. 92–101, pl. III, fig. 1.
v 1874 Valenciennesia annulata Rouss. – R. Hörnes, p. 77, pl. III, figs 1–2.
1875 Valenciennia annulata Rousseau – Sandberger, p. 701, pl. XXXII, fig. 9a. [cop. Reuss 1868]
v *1901 *Valenciennesia Arthaberi* Kramb. Gorj. – Gorjanović-Kramberger, pp. 133–134, pl. IX, figs 3–4, 6.
v 1901 *Valenciennesia Schafarziki* Kramb. Gorj. – Gorjanović-Kramberger, p. 134, pl. IX, fig. 5. [syn. nov.]
v 1901 Valenciennesia sp. n. (?) – Gorjanović-Kramberger, pl. IX, fig. 8.
v 1901 *Valenciennesia Langhofferi* Kramb. Gorj. – Gorjanović-Kramberger, pp. 134–135, text-fig. 7, pl. X, fig. 1. [syn. nov.]
v *1904 Valenciennesia Arthaberi* Kramberger-Gorjanović – Halaváts, p. 123.
*1904 Valenciennesia Langhofferi* Kramberger-Gorjanović – Halaváts, p. 124.
*1904 Valenciennesia Schafarziki* Kramberger-Gorjanović – Halaváts, p. 125.
*1923 Valenciennesia arthaberi* Gorjanović-Kramberger – Wenz, pp. 1329–1330. (cum syn.)
*1923 Valenciennesia langhofferi* Gorjanović-Kramberger – Wenz, pp. 1331–1332. (cum syn.)
*1923 Valenciennesia schafarziki* Gorjanović-Kramberger – Wenz, p. 1336. (cum syn.)
v 1923 *Provalenciennesia Langhofferi* – Gorjanović-Kramberger, p. 101, text-fig. 1a.
v 1923 *Provalenciennesia Schafarziki* – Gorjanović-Kramberger, p. 101.
v 1923 *Provalenciennesia Arthaberi* – Gorjanović-Kramberger, p. 101.
v 1944 Provalenciennesia arthaberi Kr.-Gor. – Moos, pp. 359–363, pl. XXII–XXIV, figs 14–16.
v 1967 Provalenciennesia langhofferi Gorjanović-Kramberger – Taktakishvili, p. 21, pl. II, fig. 3. [cop. Gorjanović-Kramberger 1901]
v 1967 Provalenciennesia schafarziki Gorjanović-Kramberger – Taktakishvili, p. 22, pl. II, fig. 4. [cop. Gorjanović-Kramberger 1901]
v 1967 Provalenciennesia arthaberi Gorjanović-Kramberger – Taktakishvili, pp. 22–24, pl. III, figs 1–3. [cop. Reuss 1868, Gorjanović-Kramberger 1901]
v *1974 Provalenciennesia arthaberi* (Gorjanović-Kramberger) – Milan et al., p. 130.
v *1974 Provalenciennesia Langhofferi* (Gorjanović-Kramberger) – Milan et al., p. 130.
1992 Valenciennesia cf. arthaberi Gorj.-Kramb. – Korpás-Hódi, pl. II, fig. 2.
v 2023 Provalenciennesia arthaberi (Gorjanović-Kramberger, 1901) – Neubauer, fig. 6.7: b.

###### Type locality.

Zagreb–Podsljeme (Gračani), Medvednica Mts., N Croatia (Gorjanović-Kramberger 1901).

###### Type material.

Lectotype: CNHM 5763-913 and paralectotype: CNHM 5758-908/1 (new designations). Milan et al. (1974) incorrectly designated the specimen CNHM 5763-913 as holotype of *Provalenciennesia arthaberi*; therefore, specimens CNHM 5763-913 and 5758-908/1 remained syntypes. Specimen CNHM 5763-913 is designated here as lectotype. Gorjanović-Kramberger (1901) figured three specimens, including probably CNHM 5762-912, which is thus another paralectotype.

###### Material examined.

(26 specimens) ***Lectotype***. Croatia • 1 spec., lectotype; Zagreb–Podsljeme (Gračani), Medvednica Mts., N Croatia; CNHM 5763-913. ***Paralectotype***. Croatia • 1 spec., paralectotype; Zagreb–Podsljeme (Gračani), Medvednica Mts., N Croatia; CNHM 5758-908/1. ***Holotype***. Croatia • 1 spec., holotype (by monotypy) of *Provalenciennesia langhofferi*; Zagreb–Bačun (Borje), Medvednica Mts., N Croatia; CNHM 5769-919. Serbia • 1 spec., holotype (by monotypy) of *Provalenciennesia schafarziki*; Beočin/Beocsin/Belcsény, Fruška Gora, N Serbia; SARA Pl.2489.

###### Other material.

Croatia • 4 specs; Zagreb–Podsljeme (Gračani), Medvednica Mts., N Croatia; CNHM 5758-908/2, 5759-909, 5761-911, 5764-914. • 2 specs; Zagreb–Vugrovec, Medvednica Mts., N Croatia; CNHM 1161. • 1 spec.; Novi Marof, Zagorje Basin, N Croatia; CNHM 5760-910. • 1 spec.; Gojlo-25 borehole, Sava Basin, N Croatia; CNHM 25. • 1 spec.; Selnica-7 borehole, Mura Basin, N Croatia; CNHM 28. • 2 specs; Ludbreg, Drava Basin, N Croatia; SARA Pl.2233. Serbia • 11 specs; Beočin/Beocsin/Belcsény, Fruška Gora, N Serbia; GS 1874/005/0001; SARA Pl.2487, Pl.2490, Pl.2543 to Pl.2547, Pl.6667.

###### Note.

For collectors and specimen-related information see Suppl. material 1.

###### Description.

Shell thin, smooth, convex, elongated, and flat with dense, concentric, undulated, and strong ribs. These ribs become narrower towards edges of shell. On posterior edge of shell, ribs extend at a steep to vertical angle, while on anterior part of shell, they bend back in a curved shape towards protoconch. Posterior part of shell sometimes extremely flattened and smooth (Fig. 8C). Furrows and growth lines can be observed between ribs. Dextral shell coiling, two curved whorls rapidly increasing in width and a very strongly widened inflated body whorl with an oval, elongated, and rimmed aperture. Apertural rim wide and flat and can be followed around the entire aperture. Whorls divided by wide and deep, channel-like sutures. Body whorl fully covers previous whorl and protoconch of spire. Spire displaced from posterior edge towards centre of shell. Umbo closed in adult specimens. A more pronounced folding (compared to *P. limnaeoidea*) of ribs can be observed on dextral side of shell, which is precursor of siphonal canal. Protoconch could be observed only in well-preserved laterally compacted specimens, but no such specimen is currently available. Size parameters: L = 47–153 mm, W = 37–107.5 mm, L/W = 1.15–1.85, NR = 24–26, WR = 1.5–3.3 mm, WSR = 1.2–2.5 mm.

###### Distribution and stratigraphic range.

This species was described and mentioned from some localities of old Pannonian offshore (sublittoral and profundal) marls of the Pannonian Basin: Zagreb–Podsljeme (Gračani) and Zagreb–Bačun (Borje) (Gorjanović-Kramberger 1901, 1923; this study); Zagreb–Vugrovec, Medvednica Mts. (Sremac 1981); Novi Marof, Zagorje Basin (Gorjanović-Kramberger 1901, 1923; this study); Gojlo-25 borehole, Sava Basin (Moos 1944); Selnica-7 borehole, Mura Basin (Moos 1944); Ludbreg, Drava Basin, N Croatia (this study); Beočin/Beocsin/Belcsény, Fruška Gora, N Serbia (Reuss 1868; R. Hörnes 1874; Sandberger 1875; Gorjanović-Kramberger 1901, 1923; Koch 1902; this study); Szombathely-II borehole, Danube Basin, NW Hungary (Korpás-Hódi 1992). It is a common member of the *Congeria banatica* deep-water mollusc fauna. Upper part of the *Congeria banatica* mollusc biozone. FAD of this species is ca 10.5 Ma, while its LAD is ca 9.6 Ma. *Provalenciennesia* mollusc lineage zone (ca 10.5–9.6 Ma).

###### Remarks.

*Provalenciennesia arthaberi* was originally classified as a *Valenciennesia* (= *Valenciennius*), but Gorjanović-Kramberger (1923) described *Provalenciennesia* as a separate genus and placed *P. arthaberi* into it based on the lack of well-developed siphonal canal. First mentions of this species are under the name of *V. annulatus*, but this incorrect identification was early recognised by authors (Gorjanović-Kramberger 1901). *Provalenciennesia schafarziki* and *P. langhofferi*, both described by Gorjanović-Kramberger (1901), are considered here as junior synonyms of *P. arthaberi*. Holotype specimen of *P. schafarziki* has a wide posterior flattening, smooth posterior shell surface, and its pre-siphonal structure is located in the middle part of the shell (Fig. 8C). Holotype specimen of *P. langhofferi* has the same characters as *P. arthaberi*, maybe their intercostal spaces are a little wider (Fig. 8B). This observation was also made by Gorjanović-Kramberger (1901). *Valenciennesia* sp. n. (?) figured by Gorjanović-Kramberger (1901: pl. IX, fig. 8) can be considered as a semi-adult *P. arthaberi*. He thought that it was a transitional form between *P. arthaberi* and *P. langhofferi*, but it was not described later as a separate species.

##### Provalenciennesia pauli

Taxon classificationAnimaliaLymnaeidaLymnaeidae

(R. Hörnes, 1875)

1A4125EC-8468-5268-8CE9-1CC85D48D8A7

Fig. 9A–G

v 1868 Valenciennesia annulata Rousseau – Reuss, pp. 92–101, pl. III, figs 2–3.
v 1875 Valenciennia annulata Rousseau – Sandberger, p. 701, pl. XXXII, fig. 9. [cop. Reuss 1868]
v *1875 *Valenciennesia Pauli* R. Hoern. – R. Hörnes, pp. 72–73, pl. III, fig. 1.
v 1901 Valenciennesia intermedia Kramb. Gorj. – Gorjanović-Kramberger, p. 133, text-fig. 6, pl. IX, fig. 7a–b. [syn. nov.]
v 1901 *Valenciennesia Pauli* R. Hörnes – Gorjanović-Kramberger, p. 133, pl IX, fig. 2.
*1904 Valenciennesia intermedia* Kramberger-Gorjanović – Halaváts, p. 124.
*1904 Valenciennesia Pauli* R. Hoernes – Halaváts, p. 124.
? v 1909 Valenciennesia intermedia Kramb.-Gorj. – Gorjanović-Kramberger, pl. XLVI, figs 2–2a.
*1923 Valenciennesia intermedia* Gorjanović-Kramberger – Wenz, p. 1331. (cum syn.)
*1923*Valenciennesia pauli R. Hoernes – Wenz, pp. 1332–1333. (cum syn.)
v 1923 *Provalenciennesia Pauli* R. H. – Gorjanović-Kramberger, pp. 99–100.
v 1923 *Provalenciennesia Pauli* var. intermedia – Gorjanović-Kramberger, p. 100.
1928 *Valenciennesia Pauli* R. Hörnes sp. – Pavlović, p. 37.
v 1944 Provalenciennesia pauli R. Hoernes – Moos, pp. 356–359, pl. XXII–XXIII, figs 12–13.
v 1959 Valenciennius (Provalenciennesia) pauli (R. Hoernes) – Wenz, p. 96, text-fig. 309. [cop. R. Hörnes 1875]
v 1967 Provalenciennesia pauli (R. Hoernes) – Taktakishvili, pp. 19–20, pl. II, fig. 2. [cop. R. Hörnes 1875]
v 1967 Valenciennius intermedius Gorjanović-Kramberger – Taktakishvili, pp. 47–48, pl. XI, figs 7–8. [cop. Gorjanović-Kramberger 1901]
v *1974 Provalenciennesia pauli intermedia* (Gorjanović-Kramberger) – Milan et al., p. 131.

###### Type locality.

Gornji Kneginec, Zagorje Basin, N Croatia (R. Hörnes 1875).

###### Type material.

Holotype (by monotypy): GS 1875/006/0022.

###### Material examined.

(17 specimens) ***Holotype***. Croatia • 1 spec., holotype (by monotypy); Gornji Kneginec, Zagorje Basin, N Croatia; GS 1875/006/0022. • 1 spec., holotype (by monotypy) of *Provalenciennesia pauli intermedia*; Zagreb–Šestine, Medvednica Mts., N Croatia; CNHM 5757-907/1-2.

###### Other material.

Croatia • 1 spec.; Zagreb–Podsljeme (Gračani), Medvednica Mts., N Croatia; earlier labelled as *Provalenciennesia pauli tenuicostata*; CNHM 5754-904. • 1 spec.; Zagreb–Podsljeme (Bliznec), Medvednica Mts., N Croatia; CNHM 5756-906. Serbia • 6 specs; Beočin/Beocsin/Belcsény, Fruška Gora, N Serbia; SARA Pl.2491, Pl.2548, Pl.2549; CNHM 23; GS 1874/005/0002. • 5 specs; Belgrade–Rakovica, Šumadija Hills, Central Serbia; NHMB 1863, 1864. Hungary • 1 spec.; Kisbér, Bakony Mts., W Hungary; SARA Pl.4751. • 1 spec.; Tata, Gerecse Mts., N Hungary; SARA Pl.4771.

###### Note.

For collectors and specimen-related information see Suppl. material 1.

###### Description.

Shell thin, smooth, convex, oval, and flat with dense, concentric, undulated, and strong ribs. These ribs become narrower towards edges of shell. On posterior edge of shell, ribs extend at a steep to vertical angle, while on anterior part of shell, they bend back in a curved shape towards protoconch. Furrows and growth lines can be observed between ribs. Dextral shell coiling, two curved whorls rapidly increasing in width and a very strongly widened inflated body whorl with an oval, elongated, and rimmed aperture. Apertural rim wide and flat and can be followed around entire aperture. Whorls divided by wide and deep, channel-like sutures. Body whorl fully covers previous whorl and protoconch of spire. Spire displaced from posterior edge towards centre of shell. Umbo closed in adult specimens. A more pronounced folding (compared to *P. limnaeoidea*) of ribs can be observed on dextral side of shell, which is precursor of siphonal canal. Protoconch could be observed only in well-preserved laterally compacted specimens, but no such specimen is currently available. Size parameters: L = 47–116.5 mm, W = 44–101 mm, L/W = 0.95–1.35, NR = 20–30, WR = 1.5–3.0 mm, WSR = 1.0–2.0 mm.

###### Distribution and stratigraphic range.

This species was described and mentioned from some localities of old Pannonian offshore (sublittoral and profundal) marls of the Pannonian Basin: Gornji Kneginec, Zagorje Basin (R. Hörnes 1875; Gorjanović-Kramberger 1901, 1923); Zagreb–Podsljeme (Gračani) and Zagreb–Podsljeme (Bliznec), Medvednica Mts., N Croatia (Gorjanović-Kramberger 1901, 1923); Beočin/Beocsin/Belcsény, Fruška Gora, N Serbia (Reuss 1868; Sandberger 1875; Gorjanović-Kramberger 1901, 1923; Moos 1944); Umka (Pavlović 1928); Belgrade–Rakovica, Šumadija Hills, Central Serbia (this study); Kisbér, Bakony Mts., W Hungary (this study); Tata, Gerecse Mts., N Hungary (this study). It is a common member of the *Congeria banatica* deep-water mollusc fauna. Upper part of the *Congeria banatica* mollusc biozone. FAD of this species is ca 10.5 Ma, while its LAD is ca 9.6 Ma. *Provalenciennesia* mollusc lineage zone (ca 10.5–9.6 Ma).

###### Remarks.

*Provalenciennesia pauli* was originally classified as a *Valenciennesia* (= *Valenciennius*), but Gorjanović-Kramberger (1923) described *Provalenciennesia* as a separate genus and placed *P. pauli* into it based on the lack of well-developed siphonal canal. Unfortunately, most of the well-preserved specimens are internal moulds with oval shell outline; therefore, such specimens dominate (Fig. 9). First mentions of this species are under the name of *V. annulatus*, but this incorrect identification was early recognised by authors (R. Hörnes 1875; Gorjanović-Kramberger 1901). Holotype specimen of *P. pauli intermedia* (CNHM 5757-907/1-2) is an incomplete juvenile (Fig. 12A), therefore we cannot accept as a separate species. It lacks a well-developed siphonal canal showing that it belongs to the *Provalenciennesia* genus. *Provalenciennesia pauli tenuicostata* is a nomen nudum, but its type material was found in the CNHM collection (Fig. 9C–E) and considered as *P. pauli*.

##### Provalenciennesia boeckhi

Taxon classificationAnimaliaLymnaeidaLymnaeidae

(Halaváts, 1886)

C179537B-2F42-5A8A-A0CE-E4831443EC74

Fig. 10A, B

v *1886 *Valenciennesia Böckhi* nov. sp. – Halaváts, pp. 134–135, pl. XXV, fig. 9.
1897 *Valenciennesia Böeckhi* [sic!] Halav. – Brusina, p. 2, pl. I, fig. 19.
1901 *Valenciennesia Böckhi* Halaváts – Gorjanović-Kramberger, p. 132.
v *1904 Valenciennesia Böckhi* Halaváts – Halaváts, p. 123.
*1923 Valenciennesia böckhi* Halaváts – Wenz, p. 1330. (cum syn.)
1923 *Provalenciennesia Boeckhi* Hal. – Gorjanović-Kramberger, p. 101.
v 1944 Provalenciennesia boeckhi Hal. – Moos, pp. 363–365, pl. XXIV, figs 17–18.
v 1967 Valenciennius boeckhi Halaváts – Taktakishvili, pp. 26–27, pl. IV, fig. 1. [cop. Halaváts 1886]

###### Type locality.

Ciuchici/Csukics/Tyukó, Zagajica Basin, W Romania (Halaváts 1886).

###### Type material.

Lectotype: SARA Pl.26 and paralectotype: SARA Pl.27 (new designations). *Provalenciennesia boeckhi* was described based on two specimens, but no type material was designated. Here we designate specimen SARA Pl.26 as lectotype.

###### Material examined.

(14 specimens) ***Lectotype***. Romania • 1 spec., lectotype; Ciuchici/Csukics/Tyukó, Zagajica Basin, W Romania; SARA Pl.26. ***Paralectotype***. Romania • 1 spec., paralectotype; Ciuchici/Csukics/Tyukó, Zagajica Basin, W Romania; SARA Pl.27.

###### Other material.

Romania • 2 specs; Ciuchici/Csukics/Tyukó, Zagajica Basin, W Romania; SARA Pl.2024.121.1, Pl.2024.122.1. Croatia • 1 spec.; Gojlo-25 borehole, Sava Basin, N Croatia; CNHM 33. • 2 specs; Srednji Lipovac, Požeška Gora, N Croatia; CNHM 32, 34. Serbia • 7 specs; Baćevac, Šumadija Hills, Central Serbia; NHMB 1976.

###### Note.

For collectors and specimen-related information see Suppl. material 1.

###### Description.

Shell thin, smooth, convex, oval or elongated, and flat with dense, concentric, undulated, sharp, and fine ribs. These ribs become narrower towards edges of shell. On posterior edge of shell, ribs extend at a steep to vertical angle, while on anterior part of shell, they bend back in a curved shape towards protoconch. Furrows and growth lines can be observed between ribs. Dextral shell coiling, two curved whorls rapidly increasing in width and a very strongly widened inflated body whorl with an oval, elongated, and rimmed aperture. Apertural rim wide and flat and can be followed around entire aperture. Whorls divided by wide and deep, channel-like sutures. Body whorl fully covers previous whorl and protoconch of spire. Spire displaced from posterior edge towards centre of shell. Umbo closed in adult specimens. An even more pronounced folding (compared to *P. arthaberi* and *P. pauli*) of ribs can be observed on dextral side of shell, which is precursor of siphonal canal. Protoconch could be observed only in well-preserved laterally compacted specimens, but no such specimen is currently available. Size parameters: L = 25–52 mm, W = 25–44 mm, L/W = 1.0–1.6, NR = 30, WR = 0.5–1.0 mm, WSR = 0.5 mm.

###### Distribution and stratigraphic range.

This species was described and mentioned from a few localities of old Pannonian offshore (sublittoral and profundal) marls of the Pannonian Basin: Ciuchici/Csukics/Tyukó, Zagajica Basin, W Romania (Halaváts 1886; Gorjanović-Kramberger 1901); Zagreb–Okrugljak, Medvednica Mts. (Brusina 1897); Gojlo-25 borehole, Sava Basin (Moos 1944); Srednji Lipovac, Požeška Gora, N Croatia (Moos 1944); Baćevac, Šumadija Hills, Central Serbia (this study). It is a rare member of the *Congeria banatica* and *Carinatocongeria digitifera* deep-water mollusc fauna. Uppermost part of the *Congeria banatica* and lower part of the *Carinatocongeria digitifera* mollusc biozone. FAD of this species is ca 9.8 Ma, while its LAD is ca 8.9 Ma. *Provalenciennesia* mollusc lineage zone (ca 10.5–9.6 Ma) and lower part of the *Valenciennius* mollusc lineage zone (ca 9.6–3.6 Ma).

###### Remarks.

This species is often referred to as *Valenciennius* in the literature due to its transitional character of its precursory siphonal canal. On most specimens (the best specimens are from Ciuchici/Csukics/Tyukó, see Fig. 10A, B), this canal is not well-developed; therefore, we classify it as a provalencienniid.

##### Genus Valenciennius

Taxon classificationAnimaliaLymnaeidaLymnaeidae

Rousseau, 1842

9FE90132-2475-5538-BA23-6F390867AC73

###### Synonym.

*Valenciennesia* P. Fischer, 1859.

###### Type species.

*Valenciennius annulatus* (Rousseau, 1842); original designation.

###### Original description.

“Ovate shell, transversely striated, seventeen striae with regular lines, with a curved double margin, sinistrally widened. This rare and beautiful snail, which we consider forming a new genus, is oval and covered with transverse rings. These rings appear to be points of growth and become more spaced out as the shell grows. The anterior end is curved like a hook. On the left side, there is a significant groove, which seems to be a unique organ of the animal, similar to those found in siphon-bearing gastropods. This groove is rounded and visible up to the tip of the spire. At the base of this point, there is a flat, inward part that occupies the entire front of the shell. On the opposite side of the groove, there is a small, barely noticeable bulge that disappears near the spire. This shell, which resembles an ancylinid in shape and a siphon-bearing gastropod due to the groove on the side, does not truly belong to either genus.” (in Latin and French in Rousseau 1842, translated by D. Botka).

###### Emended diagnosis.

Members of the genus *Valenciennius* are characterised by large size (≤10–15 cm in length and width), reduced coiling (1 whorl), wide oval or elongated outline and aperture, coiling present in central position, ornamentation with 2–3 mm wide, concentric, undulated, rounded or sharp ribs, siphonal canal present.

##### Valenciennius reussi

Taxon classificationAnimaliaLymnaeidaLymnaeidae

Neumayr, 1875

990C6811-87EF-5536-8952-43B7204A639C

Figs 11A–J, 12B–P

1874 Valenciennesia annulata Rousseau – Brusina, pp. 102–103.
v *1875 *Valenciennesia Reussi* Neum. nov. form. – Neumayr in Neumayr and Paul, p. 81, pl. IX, fig. 22a–c.
1878 Valenciennesia pelta Brusina – Brusina, p. 9. [syn. nov.]
1884 *Valenciennesia Reussi* Neum. – Brusina, pp. 179–180, pl. XXVII, figs 70, 72.
1884 Valenciennesia sp. – Brusina, pp. 179–180, pl. XXVII, fig. 71.
1884 Valenciennesia pelta Brusina – Brusina, pp. 180–181, pl. XXX, fig. 26.
1897 *Valenciennesia Reussi* Neum. – Brusina, p. 1, pl. I, figs 17–18.
1897 Valenciennesia pelta Brus. – Brusina, p. 2, pl. I, fig. 20.
1901 Valenciennesia krambergeri – R. Hörnes, p. 233. [syn. nov.]
v 1901 *Valenciennesia Reussi* Neum. – Gorjanović-Kramberger, pp. 130–131, text-fig. 2.
v 1901 Valenciennesia pelta Brusina – Gorjanović-Kramberger, p. 131.
v 1901 Valenciennesia alta Kramb. Gorj. – Gorjanović-Kramberger, p. 131, pl. X, figs 2–4. [syn. nov.]
v 1901 *Valenciennesia Brusinae* Kramb. Gorj. – Gorjanović-Kramberger, p. 132. [syn. nov.]
*1904 Valenciennesia alta* Kramberger-Gorjanović – Halaváts, p. 123.
*1904 Valenciennesia Brusinae* Kramberger-Gorjanović – Halaváts, p. 124.
*1904 Valenciennesia pelta* Brusina – Halaváts, p. 125.
v *1904 Valenciennesia Reussi* Neumayr – Halaváts, p. 125.
v 1909 *Valenciennesia Krambergeri* R. Hörnes – Gorjanović-Kramberger, p. 584, pl. XLVI, figs 3–4.
*1923 Valenciennesia alta* Gorjanović-Kramberger – Wenz, p. 1328. (cum syn.)
*1923 Valenciennesia brusinai* Gorjanović-Kramberger – Wenz, pp. 1330–1331. (cum syn.)
*1923 Valenciennesia krambergeri* R. Hoernes – Wenz, p. 1331. (cum syn.)
*1923 Valenciennesia pelta* Brusina – Wenz, pp. 1333–1334. (cum syn.)
*1923 Valenciennesia reussi* Neumayr – Wenz, pp. 1334–1336. (cum syn.)
v 1923 *Valenciennesia Reussi* Neumayr – Gorjanović-Kramberger, pp. 102–103, text-figs 1c, 2a.
v 1923 Valenciennesia krambergeri R. H. – Gorjanović-Kramberger, p. 103.
v 1923 *Valenciennesia Brusinai* Gorj. Kramb. – Gorjanović-Kramberger, pp. 103–104.
v 1923 Valenciennesia pelta Brus. – Gorjanović-Kramberger, p. 104, text-fig. 2b.
v 1923 Valenciennesia alta Gorj. Kramb. – Gorjanović-Kramberger, pp. 104–105.
v 1944 Valenciennius altus Kr.-G. – Moos, pp. 366–367, pl. XXIV, fig. 19.
v 1944 Valenciennius reussi Neum. – Moos, pp. 368–372, pl. XXV, figs 20–22.
v 1944 Valenciennius pelta Brus. – Moos, p. 373, pl. XXV, fig. 23.
1951 Valenciennesia reussi Neum. – Stevanović, pl. XII, figs 2–3.
1951 Valenciennesia pelta Brus. – Stevanović, pl. XII, fig. 4.
? 1951 Valenciennesia n. sp. – Stevanović, pl. XII, fig. 5.
1956 Valenciennesia pelta Brus. – Stevanović and Mladenović, pl. I, fig. 5.
v 1967 Valenciennius reussi Neumayr – Taktakishvili, pp. 34–37, pl. VII, figs 2–5. [cop. Neumayr 1875, Brusina 1884, Stevanović 1951]
1967 Valenciennius reussi pelta Brusina – Taktakishvili, pp. 37–38, pl. VII, fig. 7. [cop. Brusina 1884]
1967 Valenciennius reussi brusinai Gorjanović-Kramberger – Taktakishvili, pp. 38–39, pl. VII, fig. 6. [cop. Brusina 1884]
v 1967 Valenciennius krambergeri R. Hoernes – Taktakishvili, pp. 39–41, pl. VIII, figs 1–4, pl. IX, figs 1–4. [cop. pl. VIII, fig. 1. Gorjanović-Kramberger 1909]
v 1967 Valenciennius altus Gorjanović-Kramberger – Taktakishvili, pp. 51–53, pl. XVI, figs 2–4. [cop. Gorjanović-Kramberger 1901]
1971 Valenciennius reussi Neumayr – Széles in Góczán and Benkő, pl. IV, figs 5–6.
1973 Valenciennius reussi Neumayr, 1875 – Marinescu, p. 43, pl. XII, figs 4a–5.
v *1974 Valenciennius altus* Kramberger-Gorjanović – Milan et al., p. 139.
v *1974 Valenciennius brusinai* Kramberger-Gorjanović – Milan et al., p. 139.
v *1974 Valenciennius pelta* Brusina – Milan et al., p. 140.
*1978 Valenciennius pelto-altus* Stev. – Stevanović, p. 338. [nomen nudum]
? *1978 Valenciennius syrmicus* Stev. – Stevanović, p. 339. [nomen nudum]
1983 Valenciennius reussi Neum. – Korpás-Hódi, pl. X, fig. 6.
1990a Valenciennius reussi Neumayr – Stevanović, p. 500, pl. 15, figs 5–6.
1990a Valenciennius peltus Brusina – Stevanović, p. 500, pl. 15, figs 2–3.
? 1990a Valenciennius syrmicus Stevanović – Stevanović, pp. 500–501, pl. 15, fig. 4. [syn. nov.?]
1990 Valenciennius cf. reussi Neumayr – Stevanović and Škerlj, pl. I, fig. 15.
v 1995 Valenciennius cf. reussi Neumayr – Magyar, fig. 4: D.
v 1999 Valenciennius reussi Neumayr – Lennert et al., pl. V, fig. 4.
v 2002 Valenciennius reussi Neumayr – Gulyás et al., figs 3–6.
v 2009 Valenciennius reussi Neumayr, 1875 – Cziczer et al., fig. 6: l–m.
2013 Valenciennius reussi (Neumayr, 1875) – Katona et al., fig. 4: l.
2014 Valenciennius reussi Neumayr – Cziczer, pl. 37, figs l–p.
2016 Valenciennius reussi – Lubenescu, pl. IV, fig. 1 (a).
2016 Valenciennius krambergeri – Lubenescu, pl. IV, fig. 2 (b).
2017 Valenciennius reussi Neumayr in Neumayr and Paul – Vermeij, fig. 1: I.
? v 2020 Valenciennius reussi – Sebe et al., fig. 13.
2023 Valenciennius reussi Neumayr in Neumayr and Paul – Vinarski and Pointier, fig. 2.1: g, h.
2023 Valenciennius reussi Neumayr in Neumayr and Paul – Neubauer, fig. 6.7: c, f.
2024 Valenciennius reussi (Neumayr, 1875) – Katona, pp. 269–270, text-fig. 12: B, pl. II, fig. 15.
v 2025 Valenciennius reussi Neumayr – Magyar et al., fig. 4: k.

###### Type locality.

Kindrovo, Dilj Mts., N Croatia (Neumayr and Paul 1875).

###### Type material.

Holotype (by monotypy): GS 1875/002/0298 (a poorly preserved juvenile specimen).

###### Material examined.

(66 specimens) ***Holotype***. Croatia • 1 juvenile spec., holotype (by monotypy); Kindrovo, Dilj Mts., N Croatia; GS 1875/002/0298. • 1 spec., holotype (by monotypy) of *Valenciennius brusinai*; Zagreb–Okrugljak, Medvednica Mts., N Croatia; CNHM 5061-226. ***Neotype***. Croatia • 1 spec., neotype of *Valenciennius peltus*; Zagreb–Okrugljak, Medvednica Mts., N Croatia; CNHM 5064-229. ***Syntypes***. Croatia • 3 juvenile specs, syntypes of *Valenciennius alta*; Zagreb–Okrugljak, Medvednica Mts., N Croatia; CNHM 5066-231/1-3.

###### Other material.

Croatia • 2 specs; Bektež, Krndija Mts., N Croatia; CNHM 5747-897/1-2. • 3 specs; Bujavica-9 borehole, Sava Basin, N Croatia; CNHM 27/1-3. • 1 spec.; Ludbreg-2 borehole, Drava Basin, N Croatia; CNHM 37. • 1 spec.; Grubišno Polje-1 borehole, Sava Basin, N Croatia; CNHM 49. • 1 spec.; Zagreb–Ciglana (Črnomerec), Medvednica Mts., N Croatia; CNHM Moos-53. • 1 spec.; Zagreb, exact locality not known, Medvednica Mts., N Croatia; SARA Pl.2481. SERBIA • 4 specs; Beočin/Beocsin/Belcsény, Fruška Gora, N Serbia; SARA Pl.2488. • 2 specs; Grocka–Dubočaj, Šumadija Hills, Central Serbia; NHMB 1757. • 1 spec.; Baćevac, Šumadija Hills, Central Serbia; NHMB 1978. • 1 spec.; Mislođin, Šumadija Hills, Central Serbia; NHMB 1756. • 1 spec.; Jazovnik, Obrenovac Graben, Serbia; NHMB 1758. • 1 spec.; Ćukovine, Cer Mts., W Serbia; NHMB 1459. ROMANIA • 1 spec.; Tirol/Királykegye/Königsgnad, Banat Mts., W Romania; SARA Pl.1939. • 1 juvenile spec.; Ucuriș/Ökrös, Apuseni Mts., W Romania; SARA Pl.2013. • 1 juvenile spec.; Rădmănești/Radmanóc, Apuseni Mts., W Romania; SARA Pl.2115. Hungary • 2 specs, MOL drillcore collection; Algyő-16 borehole, Algyő High, S Hungary; SARA Pl.2024.66.1, Pl.2024.67.1. • 1 spec., MOL drillcore collection; Zákány-1 borehole, Drava Basin, SW Hungary; SARA Pl.2024.44.1. • 1 spec., MOL drillcore collection; Algyő-160 borehole, Algyő High, S Hungary; SARA Pl.2024.83.1. • 1 spec., MOL drillcore collection; Kaba-D-1 borehole, Jászság Basin, E Hungary; SARA Pl.2024.95.1. • 4 specs; Ibafa, Mecsek Mts., S Hungary; SARA Pl.2777, Pl.2778, Pl.2779. • 1 spec.; Pécs, exact locality not known, Mecsek Mts., S Hungary; SARA Pl.4112. • 2 specs; Pécs-Nagyárpád, Mecsek Mts., S Hungary; SARA Pl.4158; HNHM INV 2025.172. • 1 spec.; Liptód, Mecsek Mts., S Hungary; SARA Pl.4271. • 2 specs; Szászvár-13 borehole, Mecsek Mts., S Hungary; SARA Pl.5455. • 3 specs; Szekszárd, Szekszárd Hills, S Hungary; SARA Pl.3029, Pl.3030. • 2 specs; Sümeg, Bakony Mts., W Hungary; SARA Pl.4869. • 5 specs; Kisbér, Bakony Mts., W Hungary; HNHM INV 2025.163, INV 2025.165. • 7 specs; Tata, Gerecse Mts., N Hungary; HNHM INV 2025.166 to INV 2025.170. • 6 specs; Bátaszék, Szekszárd Hills, S Hungary; HNHM PAL 2025.10.1, INV 2025.181, INV 2025.182 to INV 2025.184, INV 2025.185.

###### Note.

For collectors and specimen-related information see Suppl. material 1.

###### Description.

Shell thin, smooth, convex, oval, and flat with dense, concentric, undulated, rounded, and strong ribs. These ribs become narrower towards edges of shell. On posterior edge of shell, ribs extend at a steep to vertical angle, while on anterior part of shell, they bend back in a curved shape towards protoconch. Furrows and growth lines can be observed between ribs. Dextral shell coiling, a single curved whorl rapidly increasing in width and a very strongly widened inflated body whorl with an oval and rimmed aperture. Apertural rim wide and flat and can be followed around entire aperture. Spire displaced from posterior edge towards centre of shell. Umbo closed in adult specimens. A well-developed siphonal canal present usually on dextral side of shell. Protoconch could be observed only in well-preserved uncompacted specimens, it is simple and smooth. Size parameters: L = 24–150 mm, W = 22.5–109.5 mm, L/W = 0.8–1.5, NR = 19–22, WR = 1.0–3.0 mm, WSR = 1.0–3.0 mm.

###### Distribution and stratigraphic range.

This species was described and mentioned from localities of young Pannonian offshore (sublittoral and profundal) marls of the Pannonian Basin: Kindrovo, Dilj Mts. (Neumayr and Paul 1875); Bektež, Krndija Mts. (Gorjanović-Kramberger 1923; this study); Zagreb–Okrugljak (Brusina 1884, 1897; Gorjanović-Kramberger 1901, 1923); Zagreb–Zvečaj, Zagreb–St. Xaver valley, and Zagreb–Kustošak (Brusina 1874); Zagreb–Ciglana (Črnomerec), Medvednica Mts. (Gorjanović-Kramberger 1923; Moos 1944); Bujavica-9 and Grubišno Polje-1 boreholes, Sava Basin (Moos 1944); Ludbreg-2 borehole, Drava Basin (Moos 1944); Bilogora Mts., N Croatia (Sokač 1990); Donja Trnova, Bobetino Brdo, Kadar, and Šatorovići, Majevica Mts., NE Bosnia-Herzegovina (Stevanović and Mladenović 1956; Stevanović 1990b); Beočin/Beocsin/Belcsény (this study); Bosanska Gradiška, Han Miladije boreholes, and Koraće-1 borehole, Sava Basin, N Bosnia-Herzegovina (Stevanović 1990b); Rožno–Srebotno, Videm-Krško Basin, SE Slovenia (Stevanović and Škerlj 1990); Čantavir/Csantavér boreholes, Čantavir Basin (Stevanović 1990c); Novi Bečej/Törökbecse boreholes, Bačka Topola Basin (Stevanović 1990c); Sremski Karlovci/Karlóca–Djubrik, Fruška Gora, N Serbia (Stevanović 1951, 1978, 1990a, 1990c); Grocka–Dubočaj, Baćevac, Boždarevac, Draževac, Vrčin, Beli Potok (tunnel), and Mislođin, Šumadija Hills, and boreholes in the Kolubara Basin, Central Serbia (Stevanović 1951, 1978, 1990a, 1990b, 1990d, 1990e; this study); Begejci boreholes, Srpska Crnja Basin (Stevanović 1990c); Jablanka boreholes, Zagajica Basin, NE Serbia (Stevanović 1990c); Zvizdar and Ćukovine, Cer Mts. (Stevanović 1990b; this study); Brdarica and Jazovnik, Obrenovac Graben, W Serbia (Stevanović 1951, 1990b; this study); Tirol/Királykegye/Königsgnad, Banat Mts. (Marinescu 1973; Marinescu et al. 1977; Vermeij 2017; Neubauer 2023; this study); Ucuriș/Ökrös and Rădmănești/Radmanóc, Apuseni Mts., W Romania (this study); Zákány-1 borehole, Drava Basin, SW Hungary (this study); Mocsa Mct-2 borehole, Danube Basin, NW Hungary (Korpás-Hódi 1983); Fábiánsebestyén-1 borehole, Fábiánsebestyén Basin, SE Hungary (Széles 1971); Kisújszállás, Endrőd, Kaba-Dél-1, Túrkeve-2, Tatárülés, Törtel-4, and Szolnok-14, -15 boreholes, Jászság Basin, E Hungary (Széles 1971); Algyő-16, -160 boreholes, Algyő High (Széles 1971; Magyar 1995; this study); Battonya and Pusztaszőlős boreholes, Battonya-Pusztaföldvár High (Széles 1971); Csikéria-3 and Üllés-ÉNy-1 boreholes, Szeged Basin (Széles 1971); Szank-1, -7 boreholes, Tázlár Basin (Széles 1971); Rém-2, -5 boreholes, Kunfehértó Basin (Széles 1971); Ibafa, Pécs, Pécs-Nagyárpád, and Liptód (Szónoky et al. 1999; this study); Kozármisleny (Katona et al. 2013); Szászvár-13 borehole, Mecsek Mts. (this study); Szekszárd, Szekszárd Hills (this study); Bátaszék, Szekszárd Hills, S Hungary (Lennert et al. 1999; Gulyás et al. 2002; Cziczer 2014); Sümeg (this study); Kisbér, Bakonyszentlászló, Pápateszér, Tapolcafő, and Devecser, Bakony Mts., W Hungary and Tata, Gerecse Mts., N Hungary (Gulyás et al. 2002; Müller et al. 2007; Cziczer et al. 2009; Katona 2024). It is a common member of the *Carinatocongeria digitifera* deep-water mollusc fauna. *Carinatocongeria digitifera* mollusc biozone. FAD of this species is ca 9.6 Ma, while its LAD is ca 3.6 Ma. *Valenciennius* mollusc lineage zone (ca 9.6–3.6 Ma). This species is originated from Lake Pannon, but it successfully migrated into the Eastern Paratethys region (Dacian Basin) in the early Pontian (Odessian). It is reported from the *Paradacna*- and *Valenciennius*-bearing deep-water lower Pontian (Upper Miocene) to Dacian and lower Romanian (Pliocene) clayey sediments (ca 6.1–2.6 Ma) (Gorjanović-Kramberger 1923; Lubenescu 2016; Popov et al. 2022).

###### Remarks.

*Valenciennius reussi* was originally confused with the Eastern Paratethyan species *V. annulatus* (Brusina 1874), but this mistake was recognised later (Brusina 1884). *Valenciennius peltus* is a junior synonym described and later figured by Brusina (1878, 1884) from Zagreb–Okrugljak. Its holotype specimen has somewhat weaker ribs and a slightly curved siphonal canal (Fig. 12B–D). *Valenciennius krambergeri* introduced by R. Hörnes (1901) bears the same characters as *V. reussi*, but it is mentioned from the Eastern Paratethyan sediments, e.g. Taman Peninsula, therefore we treat it as a junior synonym. *Valenciennius* sp. figured by Brusina (1884) is not a *P. boeckhi* specimen as stated by him later (Brusina 1897), but it is also a *V. reussi* specimen, later described as *V. brusinai* by Gorjanović-Kramberger (1901). *Valenciennius altus* and *V. brusinai* introduced by Gorjanović-Kramberger (1901) are junior synonyms as well, all of them mentioned from Zagreb–Okrugljak. The syntypes of *V. altus* are juveniles (Fig. 12H–P), while the characteristics of the holotype of *V. brusinai* (Fig. 12E–G) are not significantly different from those of *V. reussi*. Thus, we assume that the fossils from Okrugljak represent intraspecific variability and different stages of ontogeny of *V. reussi* rather than the presence of several different lymnaeid species in the same locality. Taktakishvili (1967) treated *V. peltus* and *V. brusinai* as subspecies of *V. reussi*. Stevanović (1990a) described an uncompressed juvenile specimen from Sremski Karlovci/Karlóca–Djubrik, Fruška Gora, N Serbia as *V. syrmicus*, which is probably a junior synonym of *V. reussi* as well. *Valenciennius syrmicus* is already mentioned by Stevanović (1951, 1978) as well as *V. peltoaltus*, but the latter has never been published in detail.

##### Valenciennius kiseljaki

Taxon classificationAnimaliaLymnaeidaLymnaeidae

Gorjanović-Kramberger, 1901

1501E3B8-439C-5730-BD4E-119EA827813E

Fig. 13A–E

v *1901 *Valenciennesia Kiseljaki* Kramb. Gorj. – Gorjanović-Kramberger, p. 132, text-fig. 5, pl. IX, fig. 1.
*1904 Valenciennesia Kiseljaki* Kramberger-Gorjanović – Halaváts, p. 124.
*1923 Valenciennesia kiseljaki* Gorjanović-Kramberger – Wenz, p. 1331. (cum syn.)
v 1923 *Valenciennesia Kiseljaki* Kramb. Gorj. – Gorjanović-Kramberger, pp. 107–108.
v 1944 Valenciennius kiseljaki Kr.-G. – Moos, p. 366.
v 1967 Valenciennius kiseljaki Gorjanović-Kramberger – Taktakishvili, pp. 27–28, pl. IV, fig. 2. [cop. Gorjanović-Kramberger 1901]
v *1974 Valenciennius kiseljaki* Kramberger-Gorjanović – Milan et al., p. 139.

###### Type locality.

Zagreb–Okrugljak, Medvednica Mts., N Croatia (Gorjanović-Kramberger 1901).

###### Type material.

Lectotype: CNHM 5070/235/1 and paralectotype: CNHM 5070-235/2 (new designations). *Valenciennius kiseljaki* was described based on two specimens (considered as syntypes). Specimen CNHM 5070/235/1 is designated here as lectotype.

###### Material examined.

(7 specimens) ***Lectotype***. Croatia • 1 spec., lectotype; Zagreb–Okrugljak, Medvednica Mts., N Croatia; CNHM 5070-235/1. ***Paralectotype***. Croatia • 1 spec., paralectotype; Zagreb–Okrugljak, Medvednica Mts., N Croatia; CNHM 5070-235/2.

###### Other material.

Croatia • 1 spec.; Bednja-1 borehole, Sava Basin; CNHM 35. • 2 specs; Gojlo-4 and Gojlo-17 boreholes, Sava Basin; CNHM 41, CNHM 43. • 1 spec.; Subotica-1 borehole, Drava Basin, N Croatia; CNHM 44. SERBIA • 1 spec.; Belgrade–Beli potok, Šumadija Hills, Central Serbia; NHMB 3568.

###### Note.

For collectors and specimen-related information see Suppl. material 1.

###### Description.

Shell thin, smooth, convex, elongated, and flat with dense, concentric, undulated, sharp, and fine ribs. These ribs become narrower towards edges of shell. On posterior edge of shell, ribs extend at a steep to vertical angle, while on anterior part of shell, they bend back in a curved shape towards protoconch. Furrows and growth lines can be observed between ribs. Dextral shell coiling, a single curved whorl rapidly increasing in width and a very strongly widened inflated body whorl with an oval and rimmed aperture. Apertural rim wide and flat and can be followed around entire aperture. Spire displaced from posterior edge towards centre of shell. Umbo closed in adult specimens. A well-developed siphonal canal present usually on dextral side of shell. Protoconch could be observed only in well-preserved uncompacted specimens, it is simple and smooth. Size parameters: L = 43–45.5 mm, W = 34–35.5 mm, L/W = 1.25–1.3, NR = 21–22, WR = 0.5–1.0 mm, WSR = 1.0–1.5 mm.

###### Distribution and stratigraphic range.

This species was described and mentioned from a few localities of young Pannonian offshore (sublittoral and profundal) marls of the Pannonian Basin: Zagreb–Okrugljak, Medvednica Mts. (Gorjanović-Kramberger 1901, 1923); Bednja-1, Gojlo-4, and Gojlo-17 boreholes, Sava Basin (Moos 1944); Subotica-1 borehole, Drava Basin (Moos 1944); boreholes of Ciglenica–Mrador Brdo, Moslavačka Gora, N Croatia (Basch 1990); Belgrade–Beli potok, Šumadija Hills, Central Serbia (Stevanović 1990b; this study). It is a rare member of the *Carinatocongeria digitifera* deep-water mollusc fauna. *Carinatocongeria digitifera* mollusc biozone. FAD of this species is ca 7.6 Ma, while its LAD is ca 3.6 Ma. *Valenciennius* mollusc lineage zone (ca 9.6–3.6 Ma).

###### Remarks.

*Valenciennius kiseljaki* possesses finer ribs than *V. reussi*. *Valenciennius reussi* identified by Moos (1944) from Gojlo-17 borehole is probably a semi-adult *V. kiseljaki* specimen. One of the syntype specimens (Fig. 13C–E) is a questionable fragmentary specimen with more robust ribs similar to those of *V. reussi*.

## Discussion

### An identification key of Lake Pannon lymnaeids

Building on the taxonomic revision of more than 700 fossil lymnaeid specimens, we compiled a dataset adequate for producing a practical identification key for the deep-water lymnaeid lineage of Lake Pannon (Fig. 14). Table 1 represent a taxonomic summary on the valid species and their synonyms and genera proposed not to use for Lake Pannon lymnaeids and their possible synonyms. Herein, we treat *Hiscerus* Gorjanović-Kramberger, 1923 syn. nov. as a junior synonym of *Velutinopsis* and *Undulotheca*, interpreting *Hiscerus* as representing juvenile stages of these taxa. A comparable situation is observed in *Neodelminiella* Kochansky-Devidé & Pikija, 1976 syn. nov., which represents juvenile specimens of the two *Undulotheca* species. *Velutinellus* Marinescu, 1969 syn. nov. is likewise regarded here as a junior synonym of *Velutinopsis*. We further reject the validity of *Neoclivunella* Kochansky-Devidé & Pikija, 1976 syn. nov., which most likely comprises juvenile specimens of *Valenciennius* species. By contrast, *Clivunella* Katzer, 1918 is accepted as a valid Middle Miocene taxon that is unrelated to the Late Miocene Lake Pannon fauna. Consequently, *Clivunella ovata* Gorjanović-Kramberger, 1923 and *Clivunella conica* Gorjanović-Kramberger, 1923 described from the Medvednica Mts. are not considered separate species here; instead, they most probably represent juvenile specimens of other Lake Pannon lymnaeids (*Velutinopsis*, *Undulotheca*, or *Valenciennius*). Accordingly, *Clivunella* was not present in Lake Pannon, and use of this genus should be avoided in Lake Pannon systematics.

**Table 1.** Valid species and their synonyms of the deep-water lymnaeid gastropod species of Lake Pannon. The 30 collected names represent in fact 11 clearly distinguished forms.

	Valid species	Synonyms		Valid species	Synonyms
1	*Radix croatica*		17	*Provalenciennesia limnaeoidea*	
2		*Lymnaea zelli*	18		*Provalenciennesia poljaki*
3	*Velutinopsis velutina*		19a	*Provalenciennesia pauli*	
4		*Velutinopsis simplex*	19b		*Provalenciennesia pauli intermedia*
5	*Velutinopsis rugosa*		20	*Provalenciennesia arthaberi*	
6		*Hiscerus amplectus*	21		*Provalenciennesia langhofferi*
7	*Undulotheca nobilis*		22		*Provalenciennesia schafarziki*
8		*Hiscerus undulatus*	23	*Provalenciennesia boeckhi*	
9		*Velutinopsis transiens*	24	*Valenciennius reussi*	
10		*Neodelminiella venusta*	25		*Valenciennius peltus*
11	*Undulotheca pancici*		26		*Valenciennius krambergeri*
12		*Undulotheca halavatsi*	27		*Valenciennius altus*
13		*Undulotheca rotundata*	28		*Valenciennius brusinai*
14		*Undulotheca kochi*	29		*Valenciennius syrmicus*
15		*Undulotheca gojlo*	30	*Valenciennius kiseljaki*	
16		*Neodelminiella lucinoides*			

The genus *Radix*, even the shallow-water or freshwater species, bears auriculate to high conical shell shape, well-developed body whorl and aperture, and short and acute spire (Aksenova et al. 2018). The ancestor or first member of this unique evolutionary lineage, *R. croatica*, is a relatively small-sized mollusc species with three whorls, rimmed aperture, sharp apertural edge, and inward bending columellar lip. Its general shape is very similar to other radicines, therefore it is not always easy to distinguish it from other radicines, especially if we have a poorly preserved fossil.

The genus *Velutinopsis* with two species, *V. velutina* and *V. rugosa*, shows a clearly more developed evolutionary phase, a remarkably successful adaptation to the environmental conditions of Lake Pannon. They can be found in the pelitic open-water sediments throughout the whole Pannonian Stage. They have three whorls, a thin, globose shell, and smooth shell surface (*V. velutina*) or thickened growth lines/furrows (*V. rugosa*).

Genus *Undulotheca* is also represented by two species, *U. nobilis* and *U. pancici*, and has a more reduced coiling (still located on the shell margin) with two whorls, a globose shell shape, undulated, concentric, and rounded ribs, and a simple, smooth protoconch, which is also characteristic for other lymnaeids. The two separated species are very similar, one can think that the smaller *U. nobilis* represent a juvenile stage, but it has narrow ribs and elongated outline unlike *U. pancici* which has thick ribs and an oval outline. They never occur at the same stratigraphic level or locality either.

The genus *Provalenciennesia* can be characterised by two whorls, large-sized (10–15 cm) shell, undulated ribs, with a centrally positioned coiling and a pre-siphonal structure. Four species were distinguished; *P. limnaeoidea* is a transitional form between undulothecans and provalencienniids with a coiling located in transitional position between the shell margin and centre. The other three species can be separated based on outline and rib morphology: *P. arthaberi* with elongated outline, rounded ribs, *P. pauli* with oval outline, rounded ribs, and *P. boeckhi* with fine and sharp ribs.

The genus *Valenciennius* has a very reduced coiling with one whorl in a central position, large-sized (10–15 cm) shell, undulated, concentric ribs, and a well-developed siphonal canal. Two species were distinguished based on outline and rib morphology: *V. kiseljaki*, elongated outline with fine and sharp ribs and *V. reussi*, oval outline with thick and rounded ribs.

This is the current state-of-the-art delimitations, but this identification key can be further improved in the future if more well-preserved specimens are collected or discovered in hidden museum drawers.

### Patterns of evolution

Lake Pannon molluscs were subjects of several evolutionary studies and interpretations. Species-level phyletic lineages with anagenetic evolution were recognised within the gastropod genus *Melanopsis* (Geary 1990, 1992; Geary et al. 2002) and in some cardiid bivalves (Müller and Magyar 1992; Geary et al. 2010; Magyar 2021). These evolutionary lineages consisted of subsequent chronospecies and thus did not contribute to the taxonomic diversity of molluscs in Lake Pannon. In other cases, cladogenesis via phenotypic divergence, adaptive radiation or ecological speciation was suggested for clades in *Melanopsis* (Geary 1990; Neubauer et al. 2012) and *Lymnocardium* (Magyar et al. 2016; Magyar and Katona 2023), which contributed to overall diversity. With very few exceptions, however, all these processes and events were observed in littoral settings, where changes in freshwater supply, climate, and water level might directly influence the habitats.

In the deep-water-dwelling Lake Pannon lymnaeids, both patterns occur: there seems to be a gradual morphological change at the genus level from *Radix* to *Valenciennius*, whereas some common occurrences of two genera, or two or even three species of the same genus, within a single sedimentary layer suggests cladogenetic events. Such a complex pattern is classified as anacladogenetic evolution by Stuessy et al. (1990) and Emerson and Patiño (2018). Anacladogenesis (budding speciation) describes the origin of a daughter species from a persisting ancestral species, with character-state change concentrated in the derived lineage while the ancestor remains unchanged (Parins-Fukuchi and Saulsbury 2025).

The discussed Lake Pannon lineage is possibly rooted in Central Paratethyan forms. We consider *Radix croatica* to have a Central Paratethyan origin; however, we interpret it as having been freshwater in its original habitat. Specimens similar to *R. croatica* occur in the uppermost Sarmatian limestone layers at the Pécs-Danitzpuszta section, Mecsek Mts., S Hungary (Botka et al. 2021). A very similar endemic form – *Radix kovaci* – was described from the Upper Miocene sediments of the Turiec Basin, W Carpathians, Slovakia (Neubauer et al. 2015).

### New lineage zones for Lake Pannon biozonation

Lake Pannon deposits can be traditionally divided into biozones based on their fossil content, especially mollusc and organic-walled microplankton organisms (dinoflagellate cysts and prasinophyte algae) are useful due to their rapid and spectacular evolutionary changes. Modern system of Lake Pannon biozones can be found in some recent publications (e.g. Magyar and Geary 2012; Magyar et al. 2025); however, biozonation of the profundal sediments always encountered difficulties due to low-diversity faunas and long-lived taxa (see Fig. 15).

The biostratigraphic potential of deep-water lymnaeid snails of Lake Pannon was previously emphasised by some authors (Gorjanović-Kramberger 1901, 1923; Moos 1944; Taktakishvili 1967), but the phylogenetic relationships between species was somewhat ambiguous without a careful revision of the group. In the last decades, new tools were developed for age determination of sediments and integrated stratigraphic studies yielded reliable age models in the case of some deep-water key localities, e.g. Oarba de Mureş/Marosorbó (Sztanó et al. 2005; Vasiliev et al. 2010), Beočin/Beocsin/Belcsény (ter Borgh et al. 2013), Gușterița/Szenterzsébet (Botka et al. 2019), and Pécs–Danitzpuszta (Botka et al. 2021). In addition, study of drillcore occurrences and seismic sections also contributed to the better understanding of Lake Pannon lymnaeid evolution. Based on all the available data, phylogenetic relationships and biostratigraphic importance of this group were reinterpreted and the following sublittoral-profundal mollusc biozones with estimated age intervals are proposed based on the evolution of Lake Pannon lymnaeid snails and their accompanying mollusc assemblages (Figs 15, 16). Biozone definitions follow concept and recommendations of Piller (2026).

1. **“ *Lymnocardium* ” *praeponticum* – *Radix croatica* assemblage zone**

Synonyms: “*Lymnocardium*” *praeponticum* zone (Magyar and Geary 2012), “Croatica beds” or “Croatica Formation” (Vrsaljko 1999; Sebe et al. 2020; Anđelković and Radivojević 2021), Stufe der *Radix croatica* (Moos 1944).

Interval: ca 11.6–11.4 Ma.

Base: FAD of “*Lymnocardium*” *praeponticum*.

Correlated events: Onset of Lake Pannon (brackish-water lake sediments), ^40^Ar/^39^Ar age data (11.62 Ma) from the directly underlying Sarmatian sediments in the Oarba de Mureş/Marosorbó section (Vasiliev et al. 2010), base of *Mecsekia ultima* organic-walled microplankton zone, base of *Trigonipraxis ornithopsis* littoral mollusc zone.

Top: LAD of *R. croatica*.

Correlated events: Lowermost part (C5r.2r-2n magnetozone) of the Oarba de Mureş B section based on magnetostratigraphy (modified after Vasiliev et al. 2010), top of *Mecsekia ultima* organic-walled microplankton zone, top of *Trigonipraxis ornithopsis* littoral mollusc zone.

Characteristic mollusc species: “*Lymnocardium*” *praeponticum* and other small-sized “Sarmatian-type” cardiids, *Lymnocardium winkleri*, *Paradacna cekusi*, small-sized congerians, *Radix croatica*, *Velutinopsis velutina*, *Velutinopsis rugosa*, *Corymbina* sp., *Stagnicola extensa*, *Gyraulus tenuistriatus*, *Gyraulus praeponticus*, *Gyraulus vrapceanus*, *Orygoceras* spp., small-sized hydrobiids (stress-tolerant pioneer dwarf fauna).

Typical sections: Oarba de Mureş/Marosorbó, Transylvanian Basin, Romania (Sztanó et al. 2005; Vasiliev et al. 2010) and Našice/Nekcse, Krndija Mts., Croatia (Vasiliev et al. 2007).

1. ***Velutinopsis* lineage zone (correlated to the lowermost *Congeria banatica* and *Coelogonia czjzeki* assemblage zones)**

Synonyms: *Velutinopsis* Stufe (Moos 1944).

Interval: ca 11.4–11.2 Ma.

Base: LAD of *R. croatica*.

Correlated events: Lowermost part (C5r.2r-2n magnetozone) of the Oarba de Mureş B section based on magnetostratigraphy (modified after Vasiliev et al. 2010), top of *Mecsekia ultima* organic-walled microplankton zone, top of *Trigonipraxis ornithopsis* littoral mollusc zone.

Top: FAD of *U. nobilis*.

Correlated events: Authigenic ^10^Be/^9^Be age data of the Mihalţ outcrop, Transylvanian Basin, Romania (Botka 2018), top of *Spiniferites pannonicus* organic-walled microplankton zone.

Characteristic mollusc species: “*Lymnocardium*” *margaritaceum*, *Lymnocardium winkleri*, *Lymnocardium undatum*, *Lymnocardium schedelianum*, *Paradacna cekusi*, *Paradacna syrmiense*, *Paradacna lenzi*, *Congeria banatica*, *Coelogonia czjzeki*, *Velutinopsis velutina*, *Velutinopsis rugosa*, *Gyraulus tenuistriatus*, *Gyraulus praeponticus*, *Orygoceras* spp., “*Micromelania*” *striata*.

Typical section: Našice/Nekcse, Krndija Mts., Croatia (Vasiliev et al. 2007).

1. ***Undulotheca* lineage zone (correlated to the middle *Congeria banatica* and *Coelogonia czjzeki* assemblage zones)**

Synonyms: *Undulotheca* Stufe (Moos 1944).

Interval: ca 11.2–10.5 Ma.

Base: FAD of *U. nobilis*.

Correlated events: Authigenic ^10^Be/^9^Be age data (Botka 2018), top of *Spiniferites pannonicus* organic-walled microplankton zone.

Top: LAD of *U. pancici* in the topmost part of Gușterița quarry confirmed by magnetostratigraphy (Botka et al. 2019) and in the southern part of Beočin quarry (topmost part of Beočin B section) and FAD of *P. limnaeoidea* in the Beočin white marls dated by magnetostratigraphy (ter Borgh et al. 2013).

Characteristic mollusc species: “*Lymnocardium*” *margaritaceum*, “*Lymnocardium*” *asperocostatum*, *Lymnocardium winkleri*, *Lymnocardium undatum*, *Lymnocardium schedelianum*, *Paradacna cekusi*, *Paradacna syrmiense*, *Paradacna lenzi*, *Congeria banatica*, *Coelogonia czjzeki*, *Velutinopsis velutina*, *Velutinopsis rugosa*, *Undulotheca nobilis*, *Undulotheca pancici*, *Gyraulus tenuistriatus*, *Gyraulus praeponticus*, *Gyraulus ponticus*, *Orygoceras* spp., “*Micromelania*” *striata*.

Typical sections: Gușterița, Transylvanian Basin, Romania (Botka et al. 2019) and Beočin/Beocsin/Belcsény, Fruška Gora, Serbia (ter Borgh et al. 2013).

1. ***Provalenciennesia* lineage zone (correlated to the upper *Congeria banatica* and *Coelogonia czjzeki* assemblage zones)**

Synonyms: Stufe der *Provalenciennesia* (Moos 1944).

Interval: ca 10.5–9.6 Ma.

Base: LAD of *U. pancici* in the topmost part of Gușterița quarry confirmed by magnetostratigraphy (Botka et al. 2019) and in the southern part of Beočin quarry (topmost part of Beočin B section) and FAD of *P. limnaeoidea* in the Beočin white marls dated by magnetostratigraphy (ter Borgh et al. 2013).

Top: FAD of *Valenciennius reussi*

Correlated event: Base of *Carinatocongeria digitifera* profundal mollusc assemblage zone.

Characteristic mollusc species: “*Lymnocardium*” *margaritaceum*, “*Lymnocardium*” *asperocostatum*, *Lymnocardium winkleri*, *Lymnocardium undatum*, *Lymnocardium soproniense*, *Paradacna abichi*, *Paradacna cekusi*, *Paradacna syrmiense*, *Paradacna lenzi*, *Coelogonia czjzeki*, *Velutinopsis velutina*, *Velutinopsis rugosa*, *Provalenciennesia limnaeoidea*, *P. arthaberi*, *P. pauli*, *P. boeckhi*, *Gyraulus tenuistriatus*, *Gyraulus praeponticus*, *Gyraulus ponticus*, *Orygoceras* spp., “*Micromelania*” *striata*.

Typical section: Beočin/Beocsin/Belcsény, Fruška Gora, Serbia (ter Borgh et al. 2013).

1. ***Valenciennius* lineage zone (correlated to the *Carinatocongeria digitifera* , uppermost *Coelogonia czjzeki* , *Congeria praerhomboidea* , and *Rhombocongeria rhomboidea* assemblage zones)**

Synonyms: Stufe der Valenciennien (Moos 1944).

Interval: ca 9.6–3.6 Ma.

Base: FAD of *Valenciennius reussi*.

Correlated event: Base of *Carinatocongeria digitifera* profundal mollusc assemblage zone.

Top: LAD of *Valenciennius* spp.

Correlated events: Termination of deep-water Lake Pannon deposits confirmed by seismic sections in N Serbia.

Characteristic mollusc species: *Lymnocardium hungaricum*, *L. majeri*, *L. schmidti*, *L. rogenhoferi*, *Pteradacna pterophora*, *Caladacna steindachneri*, “*Pontalmyra*” *otiophora*, *Paradacna abichi*, *P. okrugici*, *Carinatocongeria digitifera*, *Andrusoviconcha zagrabiensis*, *C. praerhomboidea*, *C. rhomboidea*, *Velutinopsis velutina*, *V. rugosa*, *Provalenciennesia boeckhi*, *Valenciennius reussi*, *Valenciennius kiseljaki*, *Radix grammica*, *Gyraulus ponticus*, *Orygoceras* spp., “*Micromelania*” spp., *Zagrabica* spp.

Typical sections: Bátaszék, Szekszárd Hills (Lennert et al. 1999) and Pécs–Nagyárpád, Mecsek Mts., Hungary (Szónoky et al. 1999) and Zagreb–Okrugljak, Medvednica Mts., Croatia (Stevanović 1990c).

## Conclusions

The investigated 700+ specimens of endemic deep-water Lake Pannon lymnaeids can be placed into five genera and eleven species. Lectotypes are designated for four species (*Provalenciennesia limnaeoidea*, *Provalenciennesia arthaberi*, *Provalenciennesia boeckhi*, *Valenciennius kiseljaki*). Despite its unusual morphological traits, Valencienniinae Gorjanović-Kramberger, 1923 syn. nov. is classified here as a synonym of Amphipepleinae Pini, 1877. We treat the following genera as synonyms: *Hiscerus* Gorjanović-Kramberger, 1923 syn. nov. of juveniles of *Velutinopsis* and *Undulotheca*, *Neodelminiella* Kochansky-Devidé & Pikija, 1976 syn. nov. of juveniles of *Undulotheca* species, *Velutinellus* Marinescu, 1969 syn. nov. of *Velutinopsis*, and *Neoclivunella* Kochansky-Devidé & Pikija, 1976 syn. nov. of juveniles of *Valenciennius*. The following species are also regarded here as synonyms: *Lymnaea zelli* M. Hörnes, 1856 syn. nov. (= *Radix croatica*), *Velutinopsis simplex* (Gorjanović-Kramberger, 1899) syn. nov. (= *Velutinopsis velutina*), *Hiscerus amplectus* (Gorjanović-Kramberger, 1901) syn. nov. (= *Velutinopsis rugosa*), *Hiscerus undulatus* (Gorjanović-Kramberger, 1901) syn. nov. (= *Undulotheca nobilis*), *Velutinopsis transiens* Moos, 1944 syn. nov. (= *Undulotheca nobilis*), *Neodelminiella venusta* Kochansky-Devidé & Pikija, 1976 syn. nov. (= *Undulotheca nobilis*), *Undulotheca halavatsi* (Gorjanović-Kramberger, 1901) syn. nov. (= *Undulotheca pancici*), *Undulotheca rotundata* Gorjanović-Kramberger, 1923 syn. nov. (= *Undulotheca pancici*), *Undulotheca kochi* Gorjanović-Kramberger, 1923 syn. nov. (= *Undulotheca pancici*), *Undulotheca gojlo* Moos, 1944 syn. nov. (= *Undulotheca pancici*), *Neodelminiella lucinoides* Kochansky-Devidé & Pikija, 1976 syn. nov. (= *Undulotheca pancici*), *Provalenciennesia poljaki* Gorjanović-Kramberger, 1923 syn. nov. (= *Provalenciennesia limnaeoidea*), *Provalenciennesia pauli intermedia* (Gorjanović-Kramberger, 1901) syn. nov. (= *Provalenciennesia pauli*), *Provalenciennesia langhofferi* (Gorjanović-Kramberger, 1901) syn. nov. (= *Provalenciennesia arthaberi*), *Provalenciennesia schafarziki* (Gorjanović-Kramberger, 1901) syn. nov. (= *Provalenciennesia arthaberi*), *Valenciennius peltus* Brusina, 1878 syn. nov. (= *Valenciennius reussi*), *Valenciennius krambergeri* R. Hörnes, 1901 syn. nov. (= *Valenciennius reussi*), *Valenciennius altus* Gorjanović-Kramberger, 1901 syn. nov. (= *Valenciennius reussi*), *Valenciennius brusinai* Gorjanović-Kramberger, 1901 syn. nov. (= *Valenciennius reussi*), *Valenciennius syrmicus* Stevanović, 1978 syn. nov. (= *Valenciennius reussi*).

In contrast to several anagenetically evolving Lake Pannon bivalve groups, the evolution of Lake Pannon deep-water lymnaeids is characterised by anacladogenetic speciation. The observed gradual increase in size, coiling reduction, and shell flattening are consistent with evolutionary trajectories documented in other gastropod groups (Neubauer 2023).

Based on the distribution data and evolutionary patterns of the studied species, the deep-water Pannonian deposits can be biostratigraphically subdivided into five units, of which four are proposed as new lineage zones in-line with the existing Lake Pannon mollusc biozonation. Biochron intervals are constrained by published and newly compiled data from outcrops, boreholes, and seismic sections. The “*L.*” *praeponticum*–*R. croatica* assemblage zone (11.6–11.4 Ma), representing the oldest known Pannonian sediments, is followed by four lineage zones defined by the first and last appearance datums (FAD and LAD) of deep-water lymnaeid taxa. *Velutinopsis* zone (11.4–11.2 Ma) is dominated by smooth-shelled, globose lymnaeids. *Undulotheca* zone (11.2–10.5 Ma) is characterised by the appearance of undulated ribs-bearing lymnaeids. *Provalenciennesia* zone (10.5–9.6 Ma) is defined by large-sized lymnaeids with reduced coiling and undulated pre-siphonal structure. The evolutionary lineage culminates in the concentric, siphonal canal-bearing representatives of *Valenciennius*, which persist through the remaining lifetime of Lake Pannon. Their long stratigraphic range supports the definition of a *Valenciennius* zone (9.6–3.6 Ma) for the sublittoral and profundal sediments of Lake Pannon.

## Supplementary Material

XML Treatment for Radix

XML Treatment for Radix croatica

XML Treatment for Velutinopsis

XML Treatment for Velutinopsis velutina

XML Treatment for Velutinopsis rugosa

XML Treatment for Undulotheca

XML Treatment for Undulotheca nobilis

XML Treatment for Undulotheca pancici

XML Treatment for Provalenciennesia

XML Treatment for Provalenciennesia limnaeoidea

XML Treatment for Provalenciennesia arthaberi

XML Treatment for Provalenciennesia pauli

XML Treatment for Provalenciennesia boeckhi

XML Treatment for Valenciennius

XML Treatment for Valenciennius reussi

XML Treatment for Valenciennius kiseljaki

## Additional information

### Artificial Intelligence (AI) use

The authors accept full responsibility for the content of the manuscript, including the disclosure of any use of AI.

The following AI tools were used in the preparation of this manuscript:

**AI tools**: Microsoft Copilot

**Used for**: Polishing the English of the main text and formatting the references and studied material sections.

### Author ORCIDs

D. Botka https://orcid.org/0000-0002-6627-4640

I. Magyar https://orcid.org/0000-0002-9236-0040

### Data availability

All of the data that support the findings of this study are available in the main text or Supplementary Information.

## Supplementary materials

Supplementary material 1 Supplementary table Data type xlsx https://binary.pensoft.net/file/1676967This dataset is made available under the Open Database License (http://opendatacommons.org/licenses/odbl/1.0/). The Open Database License (ODbL) is a license agreement intended to allow users to freely share, modify, and use this Dataset while maintaining this same freedom for others, provided that the original source and author(s) are credited. Dániel Botka, Imre Magyar
